# Discovery of Ribosomally Synthesized and Post-translationally Modified Knottins from the Deep-Sea Sponge *Stryphnus fortis*

**DOI:** 10.1021/acs.jnatprod.6c00501

**Published:** 2026-06-09

**Authors:** Lakmini N. Kosgahakumbura, Blazej Slazak, Norelle L. Daly, Rein Fadoul, Samia Mohamed, Erik Jacobsson, Ruisheng Xiong, Björn Hellman, Ulf Göransson, Chamari M. Hettiarachchi, Paco Cárdenas, Sunithi Gunasekera

**Affiliations:** † Pharmacognosy, Department of Pharmaceutical Biosciences, Biomedical Center, 8097 Uppsala University, Box 591, SE-751 24 Uppsala, Sweden; ‡ Department of Chemistry, 275456 University of Colombo, 00300 Colombo, Sri Lanka; § 86924 W. Szafer Institute of Botany, Polish Academy of Science, 46 Lubicz Street, 31-512 Kracow, Poland; ∥ Australian Institute of Tropical Health and Medicine, 91846 James Cook University, 4870 Cairns, Queensland, Australia; ⊥ Drug Safety and Toxicology, Department of Pharmaceutical Biosciences, Biomedical Center, SE-751 24 Uppsala, Sweden; # SciLifeLab, Department of Chemistry for Life Sciences, Uppsala University, Box 576, SE-75123 Uppsala, Sweden; ¶ Museum of Evolution, Uppsala University, Norbyvägen 16, SE-752 36 Uppsala, Sweden

## Abstract

Knottins are stable, structurally constrained mini-proteins with diverse bioactivities. This work presents the first report of a novel family of knottins, ‘stryphnines’, from the deep-sea sponge *Stryphnus fortis.* Peptidomic and transcriptomic analysis revealed a rich peptide repertoire and confirmed their ribosomal and sponge origin. This led to the isolation of two novel knottins containing a side-chain to side-chain ester linkage between serine 10/11 and glutamic acid 29/30, respectively, alongside one of their counterpart peptides devoid of the ester bond. The cryptic ester linkage was difficult to resolve due to β-elimination occurring at the serine side-chain ester. However, a combination of NMR, pH incubation studies, and MS/MS analysis facilitated their complete identification. Both stryphnines with and without the ester linkage exhibited no cytotoxicity and genotoxicity to mammalian cells. In contrast, stryphnines with the ester linkage strongly inhibited biofouling by barnacle larvae *Amphibalanus improvisus* and inhibited bacterial sialidase from *Clostridium perfringens*. The core knottin framework remained extremely stable in human serum at physiological conditions, despite ester bond hydrolysis occurring at high temperature and different pH treatments. The positive attributes of nontoxicity and biological stability, alongside demonstrated bioactivities, open up opportunities to further investigate the true ecological role and biosynthetic mechanisms of stryphnines.

## Graphical abstract

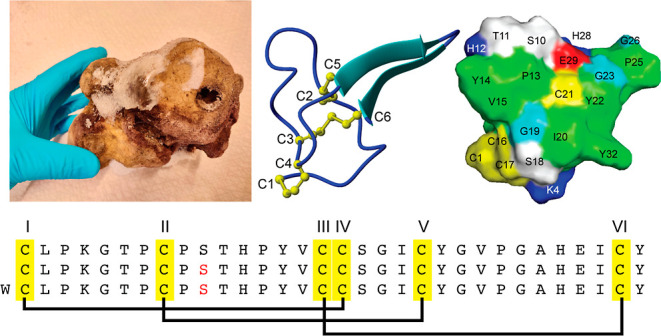

## Introduction

Cysteine-rich peptides (CRPs) marked by cross-linked multiple disulfides, typically ranging from 2 to 6 kDa, are becoming increasingly recognized as valuable mini-protein leads in drug discovery.^1–3^ Interchangeably referred to as disulfide-rich peptides (DRPs),^4,5^ CRPs offer biologically stable templates and the potential for precise targeting of historically intractable protein–protein interaction surfaces, providing many opportunities for pharmaceutical design.^6^

Among CRPs, inhibitor cystine knot (ICK) or knottins represent a unique disulfide connectivity of CysI-CysIV, CysII-CysV, and CysIII-CysVI, where the first two disulfides, along with regions of the peptide backbone, form a ring structure threaded by the third disulfide bond. This unique disulfide arrangement leads to an extremely stable peptide core.^7^ However, despite the discovery of a large number of natural products in marine sponges, including macrolides, terpenoids, nonribosomal peptides, polyketides, and sterols,^8^ the discovery of CRPs, including knottins, has remained surprisingly rare.

In the few families of CRPs identified in marine sponges, similarities and conspicuous features are prominent.^9^ Barrettides A–G from *Geodia barretti* (order: Tetractinellida, family: Geodiidae),^10,11^ stand out in this regard due to a β-hairpin scaffold held in place by two cross-braced disulfides between CysI-CysIV and CysII-CysIII. They are notable as one of the CRPs of ribosomal origin produced by sponges, validated through comprehensive transcriptomic and genomic analyses.^11,12^ The rest of the other sponge CRPs have been serendipitously discovered through bioassay-guided screening programs.^9^

Both recifin A from *Axinella* sp. (order: Axinellida, family: Axinellidae)^13^ and neopetrosiamides from *Neopetrosia* sp. (order: Haplosclerida, family: Petrosiidae) contain six cysteines but exhibit their own unique disulfide connectivity patterns, which distinguishes them from members of the knottin family.^14^ As no genomic data were obtained, their biosynthetic origin is unclear. Another peptide group characterized is the aculeines from *Axinyssa aculeata* (order: Suberitida, family: Halichondriidae),^15^ 44-residue peptides with long-chain polyamines. Their ribosomal origin was confirmed through cDNA cloning. Although suggested to be knottins, aculeines still await structural characterization and disulfide-mapping by NMR and chemical methods. Asteropsins A to G from *Asteropus* species (order: Tetractinellida, family: Ancorinidae)^16–20^ represent the only confirmed knottins identified in sponges. Most asteropsins also contain a notable posttranslational modification (PTM) of an N-terminal pyroglutamate. They represent an intriguing class of peptides, owing to their stable structural characteristics and their potential as nontoxic pharmacophore carriers. In the absence of genetic data, their biosynthetic origin remains unknown.

The close evolutionary relationship between the genus *Stryphnus* and the genus *Asteropus*,^21^ from which asteropsins are reported, inspired us to explore the production of CRPs in *Stryphnus fortis* (Vosmaer, 1885) (order: Tetractinellida, family: Ancorinidae). *S. fortis* is a massive North Atlantic deep-sea sponge,^22^ poorly studied for the presence of natural products. Stryphnusin, a brominated phenethylamine with moderate acetylcholinesterase inhibitory activity (IC_50_ – 232 μM)^23^ and ianthelline, a bromotyrosine analogue with both antifouling^24^ and cytotoxic^25^ properties, are the only two natural products extracted from *S. fortis.* Ianthelline was later shown to be most likely produced by a verongiid sponge (*Hexadella dedritifera*), growing on *S. fortis,* ^26^ which leaves stryphnusin as the only natural product potentially produced by *S. fortis* to date.

Herein, we report the characterization of a new family of stable knottins, termed stryphnines, derived from *S. fortis.* Our study employed an integrated approach of transcriptome mining and de novo peptide sequencing to unravel ∼ 21 candidate stryphnines that are of ribosomal origin and produced by the sponge. Structural characterization of the three most abundant peptides revealed a highly conserved tertiary molecular scaffold, centrally featuring a cystine-knot. The bioactivity profile of stryphnines was evaluated by screening for antibiofouling, antimicrobial, and sialidase inhibitory activities. Furthermore, their cytotoxicity and genotoxicity toward mammalian cells, stability in serum, and tolerance to heat and pH treatments were assessed. This study presents the first evidence of sponge-derived knottin-type peptides undergoing ester bond formation as a PTM, highlighting unexplored ecological roles and bioactivities that warrant further investigation.

## Results and Discussion

### Isolation and Identification of Stryphnines

To purify and isolate peptides, crude aqueous extracts of the deep-sea sponge *S. fortis* were prepared, followed by RP-HPLC, LC-MS analysis, and MS/MS sequencing. Transcriptomic and NMR analyses were conducted to facilitate the characterization of the peptides, which we subsequently coined stryphnines. The LC-MS analysis of the 60% acetonitrile (CH_3_CN) in water extract revealed ∼ 30 putative peptides, characterized by multiple charge states. Upon deconvolution, these yielded monoisotopic masses between 2.5 and 4.5 kDa (Figure S1A, Table S1). Here, we focused on the isolation of three of the most abundant peptides with monoisotopic molecular weights (MW) of 3330.39 Da, 3348.39 Da, and 3516.47 Da, later named stryphnine A [Ser10-O–CO-Glu29], stryphnine A, and stryphnine B [Ser11-O–CO-Glu30], respectively. The specific differences in monoisotopic MW of the three peptides and similar fragmentation patterns observed during subsequent MS/MS analysis suggested that these peptides may share similar amino acid sequences. The monoisotopic MW of stryphnine A [Ser10-O–CO-Glu29] was 18 Da less than that of stryphnine A, suggesting that stryphnine A [Ser10-O–CO-Glu29] may contain a PTM. The 186.087 Da difference between stryphnine A [Ser10-O–CO-Glu29] and stryphnine B [Ser11-O–CO-Glu30] suggested the presence of an additional tryptophan (W) in the sequence of the latter. This residue was assigned to the N-terminus (position 1), a configuration subsequently confirmed by 2D NMR experiments.

The peptides were reduced using dithiothreitol (DTT) and then alkylated using iodoacetamide (IAM). As a result of the alkylation, the mass of each of the three peptides increased by 348 Da, indicating the presence of six cysteine residues in each peptide (Figure S1B). MS/MS analysis of reduced/alkylated stryphnine A [Ser10-O–CO-Glu29] showed a different fragmentation pattern for the 1221.5267 *m*/*z* [M^3+^] parent ion, compared to the 1227.5278 *m*/*z* [M^3+^] parent ion of stryphnine A (Figure S1C,D). This suggested a structural divergence between the primary amino acid sequence of unmodified stryphnine A and its ester-linked variant, likely resulting from a PTM.

### *S. fortis* Transcriptome Reveals a Series of Stryphnines and Their Precursor Peptides

The *S. fortis* transcriptome, which was previously cleaned of its prokaryote sequences using a combination of polyA tail selection and in silico selection,^27^ was mined for stryphnine-like peptides, using the confirmed stryphnine A sequence. Eight stryphnine-like transcripts were identified, coding for 3–4 kDa peptides (Figure 1A). The nucleotide sequences for each of the transcripts are compiled in Table S3. Notably, none of the predicted peptide masses, except stryphnine A, were detected within the aqueous fraction. The analysis of the precursor peptides using SignalP 5.0 revealed the presence of three distinct regions: signaling, intermediate, and peptide regions. A possible cleavage site was identified between the signaling and intermediate regions (Figure 1B). The signaling peptide was identified as Sec/SPI type (secretory signal peptides transported by the Sec translocon and cleaved by signal peptidase I). This precursor peptide organization is characteristic for ribosomally produced and post-translationally modified peptides (RiPPs).^9,28^ Altogether, this is strong evidence that stryphnines are ribosomal peptides, produced by *S. fortis*, the animal host, and not by one of its microbial symbionts. There is also very little probability that the transcriptome was contaminated by animal epibionts growing on *S. fortis* (e.g., the sponge *H. dedritifera*) since the surface was avoided when subsampling for the transcriptome (A. Riesgo, Pers. comm.).

**Figure 1:**
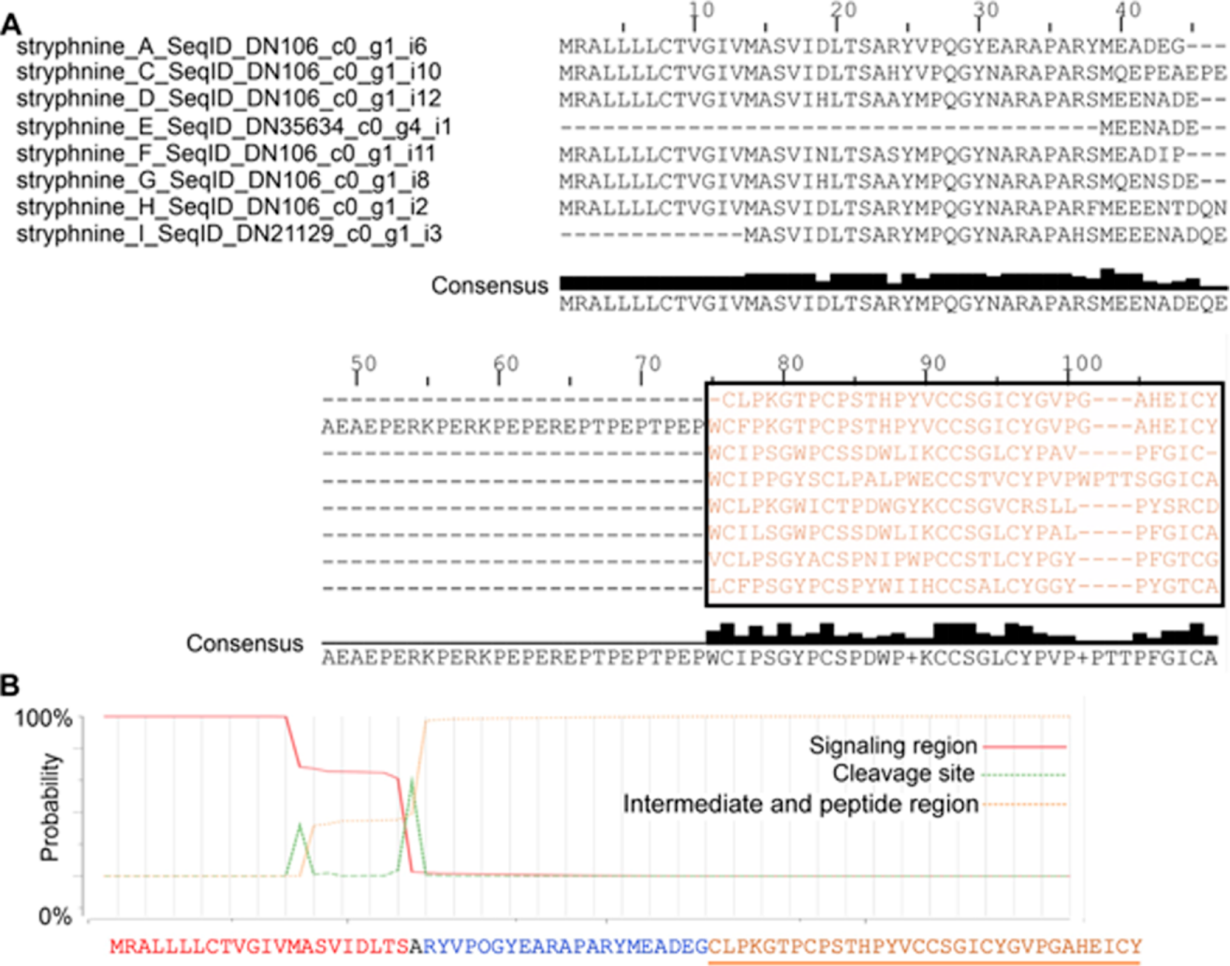
*S. fortis* transcriptome mining for stryphnine peptides and their precursors. (A) Sequence alignment of stryphnine A–I precursors, the mature peptide sequence domains are highlighted in orange and boxed in black. (B) SignalP 5.0 analysis reveals the structure of the stryphnine A precursor peptide with the secretory signal for the Sec translocon region (red) at the N-terminal, possible cleavage site (green), and intermediate region (blue) followed by the mature peptide region (in orange and underlined) at the C-terminal.

### Unravelling the Amino Acid Sequences of Stryphnines

Given the estimated size of the peptides, the presence of six cysteine residues, and the assumption that they are similar to peptides discovered in the related species *Asteropus* sp.,^17^ the *S. fortis* transcriptome was systematically searched for peptides that match these characteristics. We identified possible hits by matching the theoretical and experimental monoisotopic masses. This approach yielded a peptide with the amino acid sequence CLPKGTPCPSTHPYVCCSGICYGVPGAHEICY and theoretical monoisotopic mass of 3348.43 Da closely matching the experimental molecular weight of stryphnine A (experimental monoisotopic mass = 3348.39 Da). A potential match for stryphnine B (calculated as stryphnine A with an N-terminal tryptophan addition) was not identified in the transcriptome, despite the experimental molecular weight and the proposed sequence (WCLPKGTPCPSTHPYVCCSGICYGVPGAHEICY) being confirmed by mass spectrometry and NMR for stryphnine B with the PTM.

### MS/MS Analysis of Stryphnines

To confirm the sequences of the peptides as well as the nature of the PTM in stryphnine A [Ser10-O–CO-Glu29] and stryphnine B [Ser11-O–CO-Glu30], we undertook a series of LC-MS/MS experiments. The sequencing was challenging due to the PTM at Ser10/Ser11 in the respective peptides. The PTM cross-link proved to be pH-sensitive and susceptible to hydrolysis. Additionally, peptides underwent further chemical modification during LC-MS processing, such as N-terminal *S*-carbamoylmethylcysteine cyclization.

The chymotrypsin cleavage of alkylated stryphnine A resulted in three fragments with *m*/*z* [M^2+^] = 799.372; *m*/*z* [M^1+^] = 1102.574; and *m*/*z* [M^1+^] = 1018.379 (Figure 2A–C). The first of the three fragments, located at the N-terminal of the predicted stryphnine A sequence, apparently underwent an N-terminal *S*-carbamoylmethylcysteine cyclization in the bicarbonate buffer used for the enzymatic cleavage, resulting in a −17 Da difference.^29^ The MW and fragmentation pattern of such a modified peptide matched the predictions, and the modification could be pinpointed to the N-terminal (Figure 2A). The middle and C-terminal fragments from the chymotrypsin cleavage matched very well with the predicted mass and fragmentation patterns (Figure 2B,C). MS/MS fragmentation further verified that the C-terminus consisted of an unmodified tyrosine with a free carboxyl group (-COOH). Notably, the same middle and C-terminal fragments from chymotrypsin cleavage were obtained for stryphnine A [Ser10-O–CO-Glu29] and stryphnine B [Ser11-O–CO-Glu30]. All fragments arising from enzymatic cleavage are included in Table S2.

**Figure 2:**
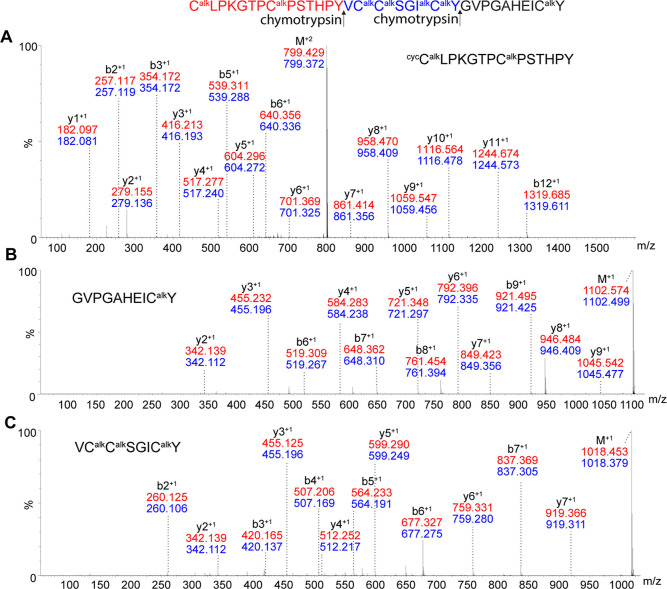
MS/MS fragmentation patterns of the three fragments of the reduced and alkylated stryphnine A resulting from chymotrypsin cleavage. (A) N-terminal fragment, (B) C-terminal fragment, and (C) middle fragment. The experimental *m*/*z* are marked with red and the theoretical with blue. The N-terminal fragment contains a cyclized N-terminal *S*-carbamoylmethylcysteine (^cyc^C^alk^). Stryphnine A [Ser10-O–CO-Glu29] cleaved with chymotrypsin gave the same two fragments as stryphnine A of *m*/*z* [M^1+^] = 1102.574 and *m*/*z* [M^1+^] = 1018.379, for middle and C-terminal fragments, respectively, and their fragmentation patterns corresponded to the predictions (Figures 2B,C). (Note that C^alk^ = C^alkylated^).

### Observation of β-Elimination at the Ser10 in Stryphnine A [Ser10-O–CO-Glu29]

The −18 Da modification could be localized to the N-terminal fragment, which, together with the cyclic N-terminal *S*-carbamoylmethylcysteine, resulted in a mass difference of −35 Da compared to the mass calculated for the non-modified sequence. This peptide fragment of *m*/*z* [M^+2^] = 790.367 resulting from chymotrypsin cleavage matched the predicted *m*/*z*, including both latter modifications (*m*/*z* [M^+2^] = 790.421) (Figure 3A). The fragmentation patterns agreed with predictions and indicated a loss of water at Ser10, consistent with the presence of a dehydrated serine residue (Figure 3A).

**Figure 3:**
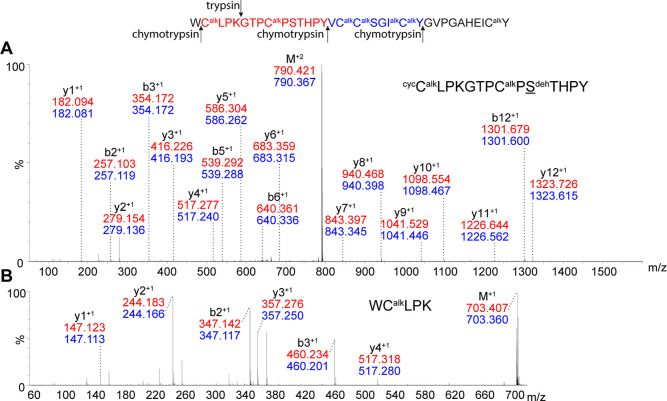
Sequencing of stryphnine peptides: the MS/MS fragmentation patterns of stryphnine A [Ser10-O–CO-Glu29] and stryphnine B [Ser11-O–CO-Glu30]. (A) The MS/MS fragmentation pattern of the N-terminal fragment of reduced and alkylated stryphnine A [Ser10-O–CO-Glu29] after chymotrypsin cleavage. Dehydration of serine (dehydroalanine) could be pinpointed to the Ser10 position. The other peptide fragments after chymotrypsin cleavage were the same as in stryphnine A. The N-terminal fragment contains a cyclized N-terminal *S*-carbamoylmethylcysteine (^cyc^C^alk^). Note that the sequence depicted on top is for stryphnine B [Ser11-O–CO-Glu30], featuring an N-terminal tryptophan to clearly illustrate all enzymatic cleavage sites common to the stryphnines. (B) MS/MS fragmentation of the N-terminal fragment of stryphnine B [Ser11-O–CO-Glu30] after trypsin cleavage shows that the difference between it and stryphnine A [Ser10-O–CO-Glu29] is the additional tryptophan (W) at the N-terminal of the sequence (Note that C^alk^ = C^alkylated^).

The chymotrypsin cleavage of alkylated stryphnine B [Ser11-O–CO-Glu30] gave the same fragments as stryphnine A [Ser10-O–CO-Glu29] because the enzyme cuts after tryptophan, on the N-terminal of the peptide. This cleaved tryptophan, a single amino acid, was too polar to be detected in the used LC-MS setup. Therefore, the alkylated peptide was cleaved with trypsin, which gave a fragment with *m*/*z* [M^+1^] = 703.360, matching the predicted *m*/*z* and fragmentation pattern and proving the presence of an additional tryptophan (Figure 3B). The MS/MS fragments after cleavage of each peptide by the enzymes chymotrypsin and trypsin are summarized in Table S2.

### Ruling Out Phosphorylation

MS/MS sequencing of the N-terminal fragments suggested the possibility of a dehydrated serine (dehydroalanine) at Ser10 and Ser11 in stryphnine A [Ser10-O–CO-Glu29] and stryphnine B [Ser11-O–CO-Glu30], respectively. However, treatment with benzyl mercaptan produced no mass increment, confirming that dehydroalanines are not present in the peptides (Figure S2). This indicated that the suggested dehydroalanines during sequencing were likely an artifact of MS/MS conditions. Furthermore, NMR chemical shifts at Ser10 and Ser11 in respective peptides did not support the presence of dehydroalanine. A literature review indicated that β-elimination can occur in phosphorylated serines, leading to a neutral loss of the phosphor group, together with a water molecule under mass spectrometry conditions,^30^ which suggested that these peptides might be phosphorylated. To test this hypothesis, a synthetic variant with phosphorylation at Ser10 was synthesized; however, its ionization pattern differed from that of the native stryphnine A [Ser10-O–CO-Glu29], suggesting that the native peptide is not phosphorylated (Figure S3A). Additionally, 1D ^31^P NMR spectroscopy showed no signals indicative of phosphorylation (Figure S3B). In contrast, treatment of both stryphnine A [Ser10-O–CO-Glu29] and stryphnine B [Ser11-O–CO-Glu30], with phosphoserine antibodies yielded positive signals (Figure S3C). It appears these antibodies gave misleading false positive signals and bound to the ester-modified peptides due to the structural and conformational similarity at Ser10/Ser11, possibly due to the O-ester at those residues. Therefore, the possibility of an ester bond at the Ser, which could be hydrolyzed during the reduction, alkylation, and enzymatic cleavage carried out under basic conditions, was proposed, and further experiments were conducted to validate this hypothesis.

### Intramolecular Ester Bond Validated by Chymotrypsin Cleavage at pH 8.0 and Shortened Incubation Time

To validate the presence of an ester bond, the chymotrypsin digestion protocol was modified, conducting digestion of stryphnine A [Ser10-O–CO-Glu29] at pH 8.0 in Tris–HCl buffer (instead of pH 8.5 in NH_4_HCO_3_ buffer). After 3 h, the resultant two fragments from chymotrypsin cleavage, which occurs after Tyr residues in the sequence, were isolated using RP-HPLC (Figure 4). One fragment corresponded to the middle fragment VCCSGICY (*m*/*z* [1+] 1018.36), while the second fragment (*m*/*z* [3+] 894.41) matched the mass of a peptide containing both N-terminal fragment CLPKGTPCPSTHPY (*m*/*z* [2+] 799.37) and C-terminal fragment (GVPGAHEICY) (*m*/*z* [2+] 551.75), with the ester bond between them remaining intact (Figure 4). This provided strong evidence of an ester bond linking the latter two fragments, which remained intact at a pH of 8.0. Incubation of this peptide sample at room temperature led to hydrolysis, yielding two distinct fragments, corresponding to N- and C-terminal fragments (Figure 4). Given the absence of other acidic amino acid side chains and the presence of an unmodified peptide C-terminus, the ester linkage was assigned between the Ser10 and Glu29 residues. To further support the presence of the ester, additional NMR experiments were carried out (outlined in the NMR results section).

**Figure 4:**
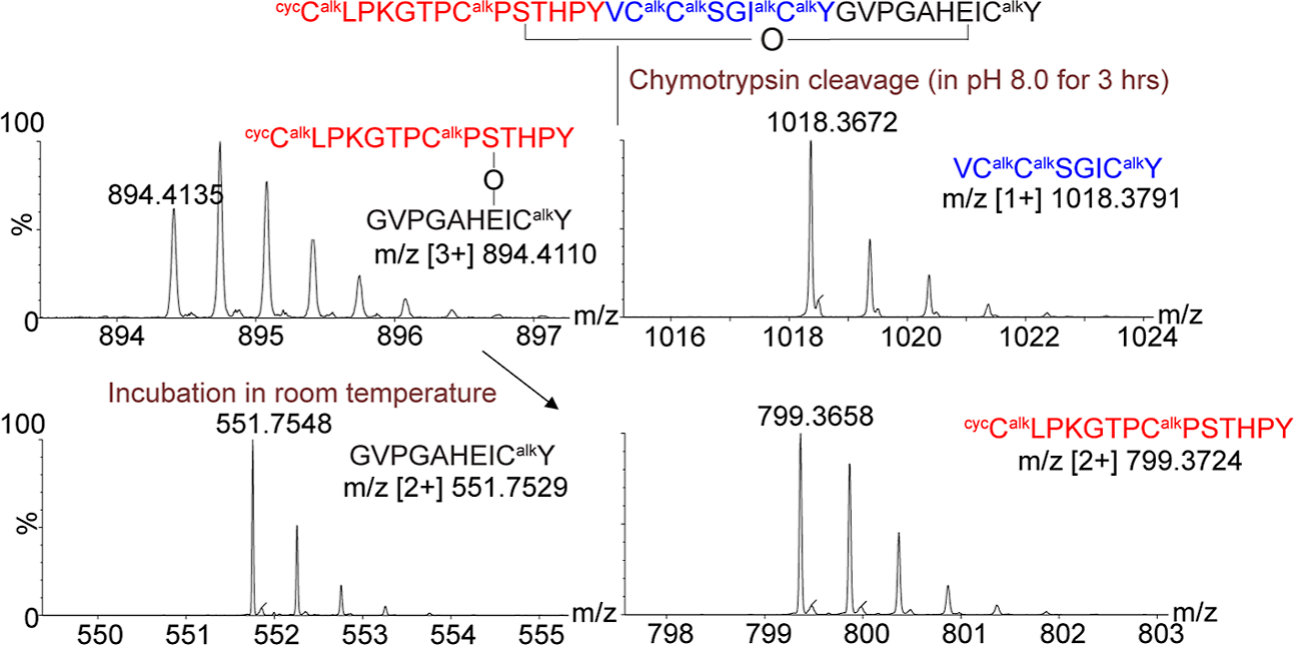
MS spectra for stryphnine A [Ser10-O–CO-Glu29] following reduction, alkylation, and chymotrypsin cleavage (under mild conditions). Reduced and alkylated (^alk^C) stryphnine A [Ser10-O–CO-Glu29], containing a cyclized N-terminal *S*-carbamoylmethylcysteine (^cyc^C) (monoisotopic peak at *m*/*z* [+3] = 1221.5267), was treated by chymotrypsin under mild conditions (Tris–HCl buffer, pH 8.0, 3 h), leading to two cleavage products. The two fragments were isolated through HPLC after the cleavage: the middle fragment and the N-terminal and C-terminal fragments linked with an ester bond [Ser10-O–CO-Glu29]. The ester cross-linked peptide sample incubated at room temperature was found to undergo hydrolysis, yielding N- and C-terminal fragments. (Note that C^alk^ = C^alkylated^)

### Synthesis of Stryphnine A

To obtain sufficient amounts for NMR analysis and bioassays, stryphnine A was synthesized by Fmoc-SPPS, oxidatively folded and confirmed to contain the native disulfide connectivity by coelution experiments by RP-HPLC and LC-MS (Figure S4A). Stryphnine A was synthesized without difficulty and presumably the incorporation of the pseudoproline dipeptide at Val^16^ and Cys^17^ positions facilitated peptide chain elongation without peptide chain aggregation (crude yield ∼ 40%). A prominent early eluting peak was evident under most of the oxidative folding conditions (Figure S4B, Table S4). From these, condition 6 was chosen for large scale folding, as shown in Figure S4B. A similar synthesis and oxidative folding protocol was employed for the synthesis of stryphnine A (*p*-stryphnine A), with the only modification being the incorporation of phosphoserine residue at position 10. *p*-stryphnine A ionized efficiently under MS conditions, exhibiting a deconvoluted monoisotopic mass of *m*/*z* 3429.44 [M + H]^1+^ (Figure S3).

### Characterization of the Ser–Glu Ester Linkage by NMR

To identify the structural basis of the −18 Da mass difference between stryphnine A and its [Ser10-O–CO-Glu29] variant, NMR analysis was performed on three related peptides: stryphnine A, stryphnine A [Ser10-O–CO-Glu29], and stryphnine B [Ser11-O–CO-Glu30]. Spectra recorded at 290 K (600 MHz, pH 5) revealed well-dispersed resonances, indicating that the peptides adopt well-defined, structured conformations in water. While most spin systems were assigned, the amide fingerprint regions of the TOCSY spectra (Figures S5 and S6) showed that Ser10 in stryphnine A variant and Ser11 in stryphnine B variant were notably difficult to observe, complicating the initial identification of the suspected Ser-O-ester.

Resolution was achieved using ^13^C HSQC and edited ^13^C HSQC spectra, where the Hβ values of Ser10 exhibited significant downfield shifts and increased differentiation compared to the unmodified peptide (Figure 5A). The use of edited HSQC allowed for the clear distinction of these downfield-shifted methylene protons at Ser10 due to their specific phase sensitivity (Figure 5C,D).

**Figure 5:**
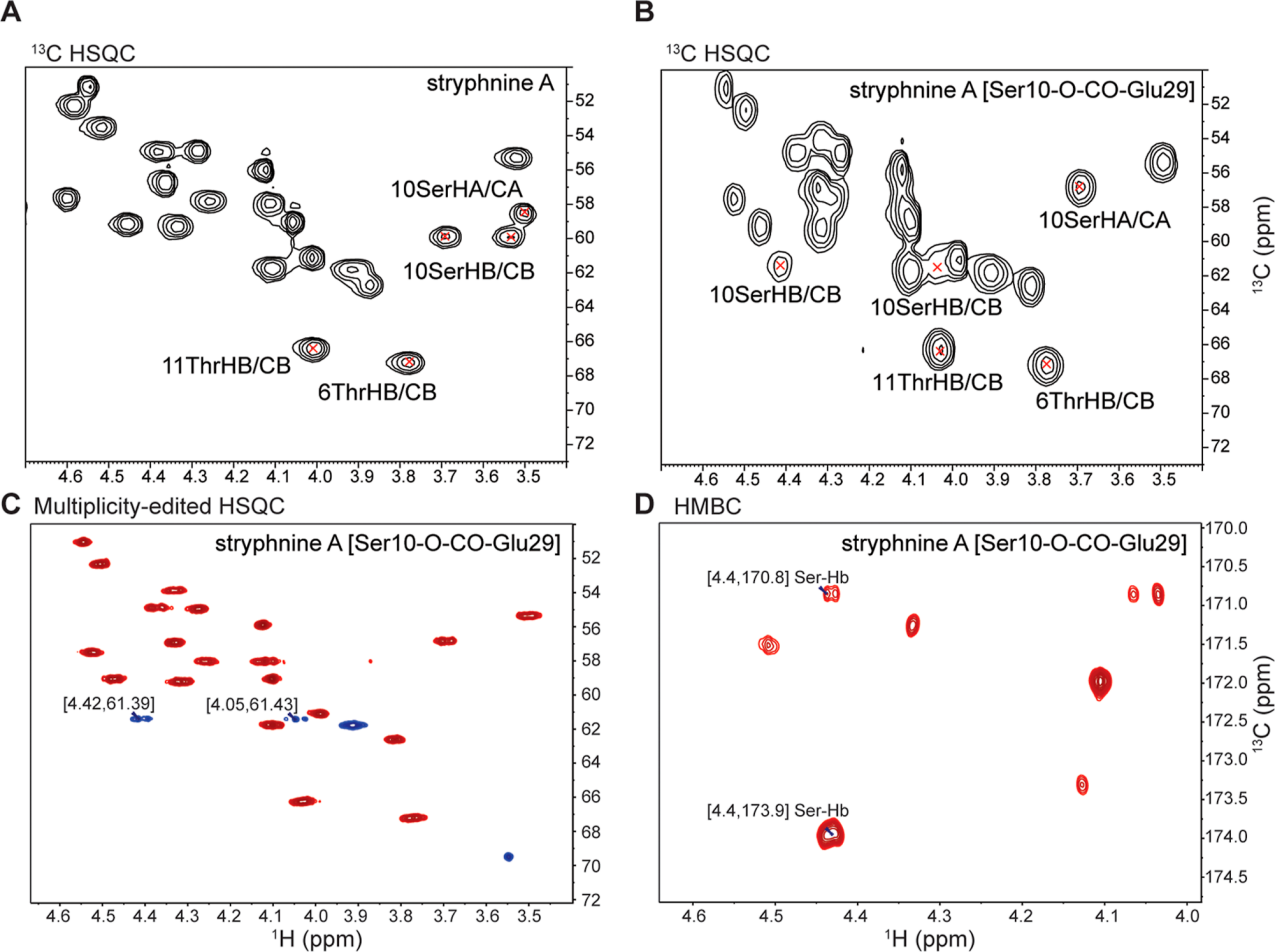
HSQC and HMBC spectra of stryphnine peptides indicating the presence of Ser-O-ester linkage. ^13^C HSQC spectrum for (A) stryphnine A and (B) stryphnine A [Ser10-O–CO-Glu29]. The HA and HB values for Ser10 in stryphnine A [Ser10-O–CO-Glu29] are deshielded downfield relative to those in stryphnine A, with a notable large separation between the two HB atoms in the ester-linked form. (C) Multiplicity edited HSQC confirms that the two HB of Ser10 (highlighted in blue) are methylene protons (with negative phase, shown in blue). (D) HMBC correlations from Ser10 demonstrate connectivity to two carbonyl groups; these peaks were assigned to the backbone carbonyl of serine (4.4, 173.9) and the side-chain carbonyl of Glu (4.4, 170.8), the latter being consistent with the formation of Ser-O-ester bond.

Final confirmation of the linkage type was provided by the HMBC spectrum, which showed long–range correlations (^2^ *J* and ^3^ *J*) between the Ser10 Cβ and two distinct carbonyl carbons (Figure 5B). While these correlations suggested either an ester or an amide bond, the pH incubation studies confirmed the presence of the ester linkage.

### NMR Assignment and Determination of Secondary Structure of Stryphnines

Sequential assignments were completed using TOCSY and NOESY data (Figures S5 and S6). Some residues (e.g., Cys1 and Lys4) and all prolines lacked TOCSY amide correlations, however, they were successfully identified via NOESY cross-peaks. Strong dαδ_(i,i+1)_ NOEs and 13C Δ_βγ_ values (<4.8 ppm for Pro3, 7, and 25) confirmed a trans configuration for all proline residues across all three peptides.

Secondary αH chemical shift analysis revealed consistent patterns among the three peptides, suggesting that the ester linkage does not significantly alter the structure and that the peptides share a very similar overall fold (Figure 6A). Secondary shifts exceeded 0.1 ppm in several regions, indicating a predominant β sheet framework interspersed with turns and helical segments (Figure 6B).

**Figure 6:**
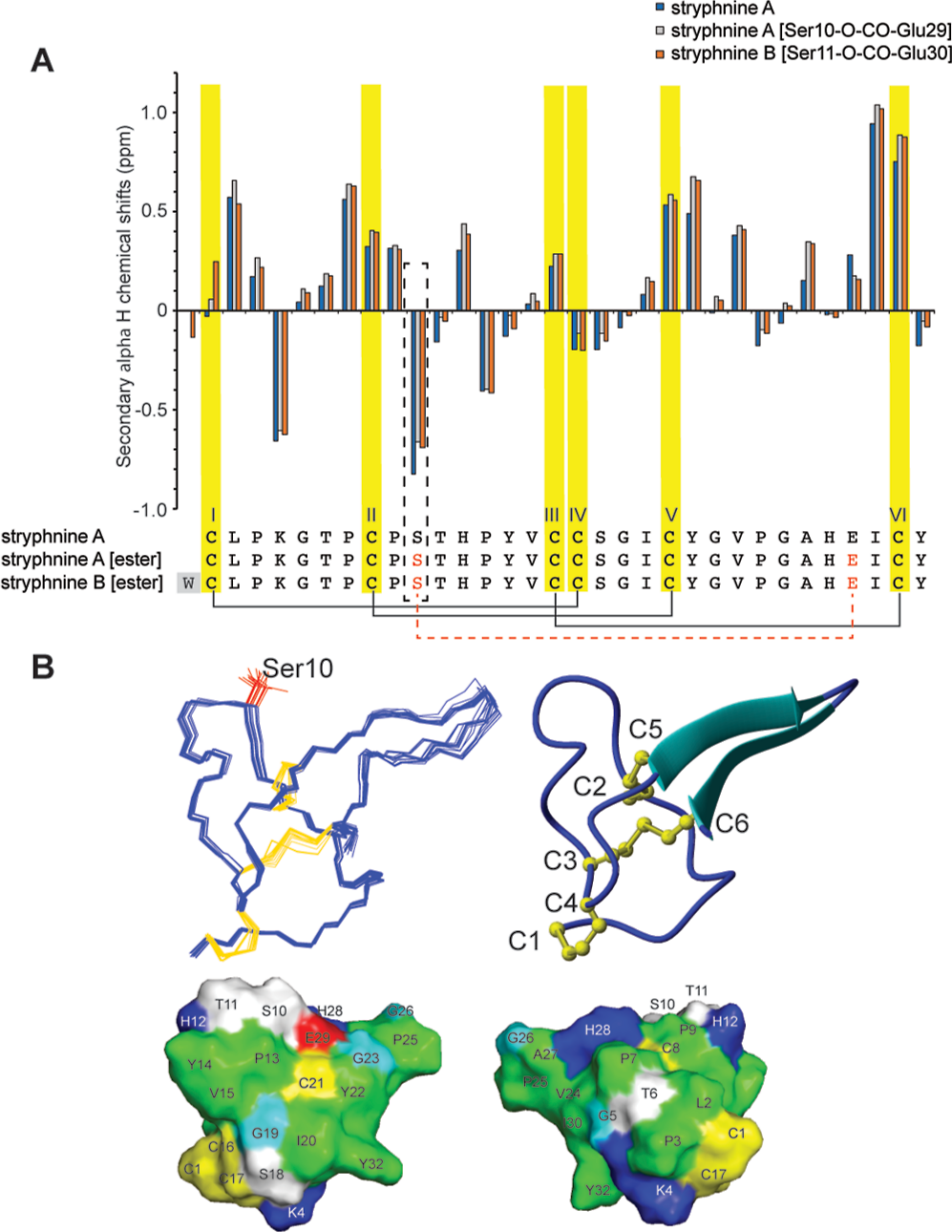
NMR analysis of stryphnine A, stryphnine A [Ser10-O–CO-Glu29], stryphnine B [Ser11-O–CO-Glu30]. (A) The αH chemical shifts of the three peptides were compared with the random coil values^31^ to obtain secondary αH chemical shifts. Ser10/Ser11 and Glu29/30 at which ester linkage occurs in stryphnine A [Ser10-O–CO-Glu29] and stryphnine B [Ser11-O–CO-Glu30] is highlighted in red within a dashed box. The knottin-type disulfide connectivity between, CysI-CysIV, CysII-CysV, and CysIII-CysVI is highlighted in yellow. (B) The three-dimensional structure of stryphnine A determined by NMR spectroscopy and the surface representation of the lowest energy structure of stryphnine A. Superposition of the 20 lowest energy structures of stryphnine A from Cys1 to Tyr32 and the ribbon representation of the lowest energy structure. Different color coding was used for the negatively charged (red), hydrophobic (green), polar (white), cysteine (yellow), and glycine (cyan) residues.

### Three-Dimensional Structure of Stryphnine A

Although we assigned the chemical shifts for all isolated peptides, the structural calculations were performed using synthetic stryphnine A. This approach was preferred because the synthetic peptide was obtained in higher quantity and greater purity than natural isolation, facilitating clearer assignments and a greater number of constraints to define the structure. The three-dimensional solution structure of stryphnine A was determined using an ensemble of the 20 lowest-energy structures, which exhibited high-quality covalent geometry and strong agreement with experimental data (Figure 6B, Table 1). The fold is stabilized by a dense network of hydrogen bonds, identified by low temperature coefficients (≤4.6 ppb/K) and slow solvent exchange (Table S5), and a characteristic ICK motif, similar to that of other known knottins, including asteropine A and asteropsins.^16–20^ The disulfide connectivity (CysI-CysIV, CysII-CysV, and CysIII-CysVI) was confirmed as the most energetically favorable arrangement, with no evidence of disulfide isomerization between 285–300 K (i.e., Hβ atoms of the cysteine residues remained nondegenerate and maintained disparate shift differences). Structurally, the peptides feature a typical C-terminal β-hairpin and a distinct surface distribution where hydrophobic residues cluster together centrally, flanked by polar and charged residues. Notably, the final 3D-fold places the side chains of Ser10 and Glu29 in close spatial proximity, providing the structural basis for the formation of the internal ester bond. The disulfide bonds are primarily buried in the center of the molecule, with the exception of Cys1-Cys17, which has some surface exposure.

**Table 1:** NMR Structural Statistics of Stryphnine A

NMR distance restraints and dihedral constraints	stryphnine A
interproton distance restraints	444
intraresidue, |*i* – *j*| = 0	89
sequential, |*i* – *j*| = 1	149
medium range, 1 < |*i* – *j*| < 5	75
long range, |*i* – *j*| ≥ 5	131
disulfide-bond restraints (6 restraints per bond)	18
dihedral-angle restraints	35
hydrogen bond restraints (4 restraints per bond)	20
R.m.s. deviations from mean coordinate structure (Å)	
backbone atoms	0.33 ± 0.14
all heavy atoms	0.84 ± 0.21
Ramachandran statistics	
% in most favored region	82.4
% residues in additionally allowed regions	17.6

Despite the close phylogenetic relationship between *S. fortis* and *Asteropus* sp.,^21^ none of the unique structural features of the asteropsin family, including high anionic amino acid content or the presence of multiple *cis* proline residues, were found in stryphnines.^19^ The peptides described here consist of five proline residues, all of which are in the trans configuration.

### The Knottin Scaffold of Stryphnine Is Stable under Elevated Temperature, Variable pH Conditions, and in Human Serum

Stryphnine A remained stable under both acidic and neutral pH conditions at elevated temperature (70 °C), maintaining its structure throughout a 1 h incubation. In contrast, both stryphnine A [Ser10-O–CO-Glu29] and stryphnine B [Ser11-O–CO-Glu30] irreversibly underwent an increase in 18 Da, in both acidic and neutral pH conditions at elevated temperature (Figure S7A,B). The ester-hydrolyzed stryphnine A [Ser10-O–CO-Glu29] was isolated by RP-HPLC, and its coelution with stryphnine A confirmed its identity as the same compound (Figure S7C). NMR analysis also revealed that the resulting ester-hydrolyzed peptide is in fact stryphnine A (data not shown). Thus, the Ser-O-ester bond appeared sensitive to hydrolysis. Nevertheless, the structural integrity of the scaffold remained intact under these conditions, as evidenced by a consistent mass corresponding to the ester-hydrolyzed peptide. Comparatively, the physiological stability of all stryphnines in serum at 37 °C was much more consistent, as all three peptides showed their intact mass over a 24 h incubation period (Figure S7D). The extensive hydrogen-bonding network identified in the amide-coefficient NMR experiments (Table S5) reinforces the core fold and could underlie the observed high stability of the core structure. Nevertheless, it is noteworthy that none of the stryphnines was stable at very high pH and temperature (pH 13 and 70 °C), as no intact peptide masses were detected at these conditions.

### Stryphnines Prevent Biofouling of *Amphibalanus improvisus* Larvae

The antifouling potential of stryphnine A [Ser10-O–CO-Glu29] and stryphnine B [Ser11-O–CO-Glu30] was evaluated against the settlement of *A. improvisus* (bay barnacle) cyprid larvae. Both peptides exhibited a concentration-dependent inhibition of larval settlement over a three-day incubation period. Notably, stryphnine B [Ser11-O–CO-Glu30] demonstrated potent activity, reducing settlement to 11 ± 3% and 6 ± 3% at concentrations of 0.2 μM and 2 μM, respectively, compared to the 86 ± 3% settlement observed in the control (Milli-Q water) (Figure 7). While stryphnine A [Ser10-O–CO-Glu29] showed a comparatively moderate inhibitory effect of 43 ± 5% and 25 ± 4% of settlement at 0.2 μM and 2 μM, respectively (Figure 7), these results align with the known activity of other β hairpin-containing marine sponge peptides, such as barrettide A, B, and C.^10,11^ These data suggest that stryphnines likely function as a selective defense mechanism for the sponge *S. fortis*, effectively deterring common foulers like barnacles, though this protection does not appear to extend to certain specialized epibionts such as the sponge *H. dedritifera*.^26^

**Figure 7:**
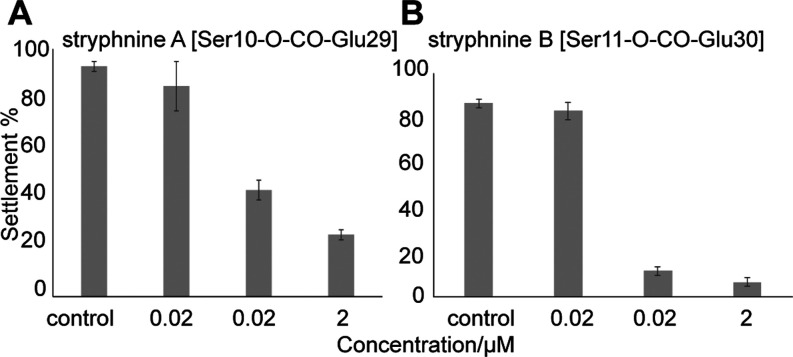
Impact of stryphnine A [Ser10-O–CO-Glu29] and stryphnine B [Ser11-O–CO-Glu30] on *Amphibalanus improvisus* cyprid larvae settlement. (A) Exposure to stryphnine A [Ser10-O–CO-Glu29] resulted in moderate inhibition of barnacle settlement. At a concentration of 0.2 μM, the settlement value was 43 ± 5%, while a higher concentration of 2 μM led to a further reduced settlement of 25 ± 4%. (B) Stryphnine B [Ser11-O–CO-Glu30] showed a more pronounced inhibitory effect on barnacle settlement, with settlement values of 11 ± 3% and 6 ± 3% at 0.2 and 2 μM, respectively. In the absence of peptide treatment (control), 93 ± 2% of the barnacles settled after 3 days of incubation in seawater. Data are given as mean values ± SD, based on three independent experiments (*n* = 3).

### Stryphnines Are Potent Inhibitors of Bacterial Neuraminidase

Building on previous reports of sialidase inhibition by structurally related knottins like asteropine A,^16^ we evaluated the stryphnines as potential inhibitors of bacterial neuraminidase (Table 2). Using an Amplex Red fluorometric assay, initial screenings at 100 μM revealed that all three peptides achieved >80% inhibition. Subsequent concentration–response studies highlighted a significant disparity in potency between the native and ester-linked forms. Table 2 and Figure S8 show that a concentration of 15 μM of stryphnine A [Ser10-O–CO-Glu29] resulted in >80% neuraminidase inhibition over a 2 h period. In contrast, stryphnine A showed weak inhibitory activity at the same concentration. Additionally, at a concentration of 9 μM, stryphnine A [Ser10-O–CO-Glu29] showed an 86% inhibition within 1 h and approximately 60% inhibition after 2 h. Notably, a 6-fold higher concentration of stryphnine A (60 μM) was required to achieve a comparable effect. This stark difference in inhibitory activity suggests that the internal ester linkage significantly enhances the peptide’s interaction with the enzyme. The IC_50_ of stryphnine A [Ser10-O–CO-Glu29] is <9 μM, indicating that it is a potent bacterial neuraminidase inhibitor, and further experiments are required to obtain a precise determination of the IC_50_ for the compounds.

**Table 2:** Neuraminidase (Sialidase) Inhibitory Activity of the Peptides^a^

compound	concentration (μM)	AR% after 30 min	AR% after 60 min	AR% after 90 min	AR% after 120 min
stryphnine A	100	91.5	91.3	91.6	93.4
stryphnine A[ester]	100	83.1	87.9	90.6	92.6
stryphnine B [ester]	100	79.4	84.1	84.9	84.1
stryphnine A [ester]	30	83.6	88.6	85.3	85.1
stryphnine A[ester]	21	86.6	94.3	89.5	91.2
stryphnine A [ester]	15	89.6	87.5	81.1	84.2
stryphnine A [ester]	9	94	86.4	65.3	57.9
stryphnine A	60	88.1	76.1	63.2	64
stryphnine A	45	64.2	45.5	37.9	43
stryphnine A	30	17.9	22.7	17.9	29.8
stryphnine A	15	0	13.6	11.6	23.7

a % reduction in absorbance (AR) at 571 nm for the neuraminidase enzyme incubated with the stryphnines was calculated relative to the absorbance of the negative control.

The observed sialidase inhibitory and antifouling activities suggest that these peptides may play a role in preventing pathogen adhesion. Sialic acids frequently serve as molecular ‘handles’ for viruses, bacteria, and neighboring cells to recognize and bind to a host. By inhibiting the enzymatic processing of these glycans or physically disrupting the settling of microorganisms, these peptides may contribute by preventing surface colonization. Although the preliminary findings from the current in vitro bioassays are promising, it remains speculative whether these bioactivities translate into a functional ecological advantage. Further studies are required to analyze their efficacy against pathogens in their native ecological settings.

### Determination of Genotoxicity and Cytotoxicity of Stryphnines

Using a cytokinesis-block micronucleus assay^32^ in CHO K1 cells, we measured both genotoxicity and cytotoxicity of stryphnines simultaneously (Figure 8). In this assay, binucleated cells (BNC) with micronuclei (MN) are formed due to a chromosomal or spindle damage in the interphase state of the cells.^33^ All tested peptides behaved similarly to the negative control, with the frequency of BNC containing micronuclei remaining at baseline levels (∼5%) even at the highest tested concentrations. Furthermore, the peptides showed no significant interference with cell proliferation, maintaining <5% cytotoxicity up to 40 μM, whereas the positive control (mitomycin C) induced significantly higher levels of damage and cell death. Similar to stryphnines, asteropine A and asteropsin A–G are reported to be nontoxic to tested cell lines.^16–20^ Although the lack of cytotoxicity to mammalian cells and high serum stability are promising attributes, further studies are necessary to evaluate the suitability of the stryphnine scaffold for potential in vivo biomedical applications.

**Figure 8:**
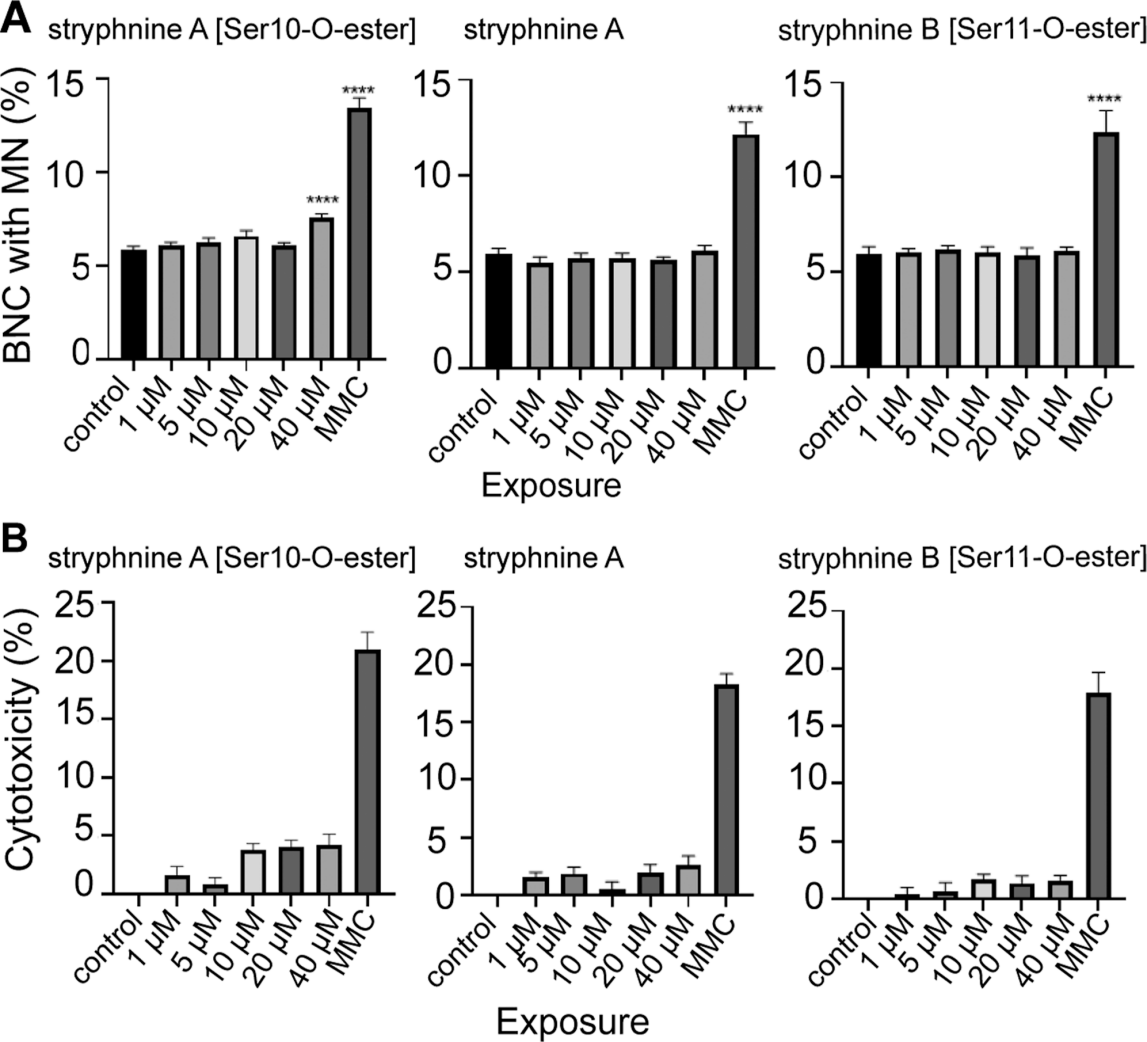
Genotoxicity and cytotoxicity of stryphnines against CHO-K1 cells. (A) Genotoxicity was assessed by the percentage of binucleated cells with micronuclei when CHO-K1 cells are exposed to different concentrations of stryphnine A [Ser10-O–CO-Glu29], stryphnine A, and stryphnine B [Ser11-O–CO-Glu30]. Data are given as means ± SEM (*n* = 9, ****p < 0.001 versus the negative control). (B) Cytotoxicity was determined by the ability to induce cell proliferation of each peptide. This was measured by comparing the cytokinesis blocker proliferation index (CBPI) of the peptides (stryphnine A [Ser10-O–CO-Glu29], stryphnine A, and stryphnine B [Ser11-O–CO-Glu30]) in the above concentrations and control cultures as a percentage.

### Determination of Antimicrobial Activity of Stryphnines

Marine sponges are known to harbor peptides with a broad range of antimicrobial activities.^34^ Thus, a two-step microdilution assay^35^ was conducted to test the antimicrobial effect of the three peptides against the two bacterial strains, *Escherichia coli* and *Staphylococcus aureus*, and the fungal strain *Candida albicans*. None of the peptides showed antimicrobial properties up to 50 μM. The human antimicrobial peptide LL37 used as the positive control, showed a potent antimicrobial activity with a minimum inhibitory concentration value between 1 and 2 μM against all the three strains. In previous reports, barrettides A and B have also not shown detectable antibacterial activity up to 80 μM against both *E. coli* and *S. aureus*.^10^ Despite these results, the potential antimicrobial activity of stryphnines cannot be discounted as our study utilized clinical pathogens and bioassay conditions do not represent ecological conditions. As a future perspective, it would be insightful to screen stryphnines against marine-derived bacterial strains that are more relevant in the context of marine sponges.

### Ester-Linked PTMs in Marine Peptides and Their Producers

Ester linkages in marine peptides are not uncommon, and several aspects have been thoroughly reviewed.^36,37^ Many of the ester-bonded peptides are derived from nonribosomal biosynthesis, such as depsipeptides or cyclodepsipeptides, where the ester linkages substitute one or more amide bonds in the backbone.^38^ Additionally, peptides containing ester linkages, e.g., callipeltin A and B,^39^ theopaupamides,^40^ jaspamides,^41^ discodermins,^42^ microviridins,^43^ cyanopeptolins,^44^ and cryptophycins,^45^ have been isolated from a number of sponges and/or their symbiotic bacteria and cyanobacteria.

Graspeptides are a diverse family of bacteria- and archaea-derived RiPPs, characterized by side chain-side chain linkages, omega-esters, and omega-amides. The omega esters and amides are installed by ATP-grasp enzymes, which are commonly co-expressed with the peptide precursor from the same bacterial gene cluster (BGC). Omega-esters are formed between the side chains of a nucleophilic residue (Thr or Ser) and an acidic residue (Glu or Asp). The nucleophilic residue is normally situated first in the sequence, counting from the N-terminal.^46^ This is also true for the stryphnines discussed in this paper: a Ser in positions 10/11 and a Glu in positions 29/30. No grasp enzymes could be found in the eukaryote sponge transcriptome, which could explain the ester bond. The precursor peptides lack the common recognition motifs for known Grasp-enzyme processing.^47^ It should still not be ruled out that new types of such enzymes could be present among the complex mixtures of symbionts present in sponges.

Another possibility for the presence of the ester bond is autocatalytic ester bond formation, where Ser 10/11 is positioned in close proximity to the Glu 29/30 residue in space, held together by the structure. This is analogous to the amide spontaneously formed to stabilize cell–surface adhesion proteins in Gram-negative bacteria.^48^ The ester bond formation could then be autocatalyzed with the aid of His residues in the vicinity of the Ser.

Detailed structural studies of the mechanics of ester bond formation in this unique peptide should be undertaken.

## Conclusions

In conclusion, we successfully characterized a novel family of structurally stable linear knottin peptides in *S. fortis*, thereby enriching the diversity of known ribosomal peptides derived from sponges. The peptides feature a rare ester linkage previously unreported in sponge-derived peptides, raising intriguing questions regarding whether this modification is catalyzed by sponge-encoded or a bacterial symbiont enzyme. Although the precise ecological role of these peptides remains elusive, their antifouling and sialidase inhibitory activities provide clues into the sponges’ potential defense mechanisms. The latter properties, coupled with lack of genotoxicity and cytotoxicity, amenability to chemical synthesis, and stability in serum, highlight that stryphnines are noteworthy of further exploration of their potential applications. Furthermore, the vast diversity of sponge species, estimated at over 9600,^49^ and the limited number of characterized ribosomal peptide families in only a handful of demosponges, underscores the vast and largely untapped potential for ribosomal peptide discovery in sponges.

## Experimental Section

### General Experimental Procedures

A specimen of *S. fortis* (class: Demospongiae, order: Tetractinellida, family: Ancorinidae) was collected by the Research Vessel *Kristine Bonnevie* (University of Bergen, Norway) with an Agassiz trawl at station KB 32 (59.5754, 4.6754), off the Hordaland shelf, Western Norway, at a depth of 224–230 m, on the 29th of April 2017. The specimen was identified based on external morphology and spicule set by sponge taxonomists H. T. Rapp and P. Cárdenas. The specimen was covered with the yellow encrusting demosponge *H. dedritifera* (order: Verongiida, family: Ianthellidae), as is commonly the case for *S. fortis*.^26^ Specimen was frozen on board and shipped frozen to Uppsala University until processing. A voucher preserved in ethanol 96% and a spicule slide were deposited in the zoological collections of the Museum of Evolution, Uppsala University, Sweden, under the museum number UPSZMC 184353.

### Extraction and Isolation of Peptides

Freeze-dried sponge material (∼250 g) was extracted to 60% acetonitrile (CH_3_CN), and the extract (∼10 g) was concentrated in a vacuum to obtain a viscous concentrate of the extract. The extract was fractionated using ÄKTA FPLC (Amersham Pharmacia Biotec) fitted to a Biotage C18 spherical flash column (100 Å, 40 g, 40–60 μm) with UV detection at 254 nm using a gradient from 100% H_2_O (0.1% trifluoroacetic acid (TFA)) to 100% CH_3_CN (0.1% TFA) over 60 min at a flow rate of 10 mL/min. Fraction collection was performed every 60 s. Fractions were analyzed by (+)-LRESI-MS using a Thermo Finnigan LCQ, and fractions containing similar molecular ion peaks were combined. The combined fractions were further purified using semi-preparative RP-HPLC with a Jupiter C18 column (250.0 × 10.0 mm 5 μm, 300 Å) at a flow rate of 3 mL/min for 70 min using a linear gradient from 5 to 75% CH_3_CN (0.05% TFA). Analytical RP-HPLC to determine the purity of the pure fractions was carried on a Kinetex XB-C18 column (250.0 × 4.6 mm, 5 μm, 100 Å) using a linear gradient from 5 to 97% CH_3_CN (0.05% TFA). The accurate mass of each pure peptide was analyzed using an Xevo G2-XS quadrupole time-of-flight (QToF) mass spectrometer coupled with a nano-Acquity UPLC (Waters Corp. Milford, MA, USA).

### Transcriptome Mining

The *S. fortis* transcriptome (GenBank SRX6964814) was obtained by Plese et al.^27^ from a specimen also collected in Western Norway, but closer to the coast (Leirvik, Hardangerfjord, 59.9813, 5.3766, 97–332 m depth, voucher 2016.09.08-St2-15). First, a search for possible sequences was undertaken. An assumption was made that the peptide in question may be similar to peptides discovered in related species (with the conserved pattern of six cysteines). The FUZZPRO program from the EMBOSS package^50^ was used to search for all peptides matching a general pattern based on the presumed similarity of the *S. fortis* peptides to asteropsins discovered in the *Asteropus* sp.:^17^ {C}­(0,3)­C­{C}­(6,6)­C­{C}­(3,4)­{C}­(0,7)­CC­{C}­(3,6)­C­{C}­(4,4)­{C}­(0,8)­C­{C}­(0,5). In this way, a database containing all possible sequences fitting the pattern, present among all ORFs predicted from the transcriptome, was generated. Subsequently, the theoretical monoisotopic MW of all of the peptide sequences in the database was calculated using a Perl script. The possible hits were identified by matching the theoretical and the LC-MS experimental monoisotopic MW.

### Sequence Analysis

To obtain the amino acid sequence, peptides were first reduced using dithioerythritol in 8 M guanidine hydrochloric acid in 1 M tris–hydrochloric acid EDTA buffer (pH 8.5) and was incubated at 37 °C in dark for 2–3 h and subsequently alkylated using iodoacetamide (50 mg in dissolved in 1 mL of 0.5 M tris acetate, 2 mM EDTA) to alkylate the free thiol groups. The alkylation reaction was quenched by the addition of 200 μL of 0.5 M citric acid after 10 min. The reduced and alkylated peptides were purified by passing through a PD10 size exclusion column. Samples were diluted to 10% CH_3_CN prior to LC-MS/MS analysis. Enzymatic cleavage of peptides, either trypsin or chymotrypsin dissolved in 50 mM NH_4_HCO_3_ buffer (pH 7.8) and incubated overnight, resulted in shorter fragments of the pure peptide. The sequence analysis was performed using nanospray MS-MS based on the b and y ion series and was confirmed with the sequences mined from the transcriptome.

To characterize the ester bond, stryphnine A­[Ser10-O-ester] (0.5 mg) was fully reduced using 3.9 mM DTT in a 1 M Tris–HCl (pH 8) and 8 M GnHCl (pH 6.6) buffer mixture (1:3 ratio), resulting in a final reduction buffer pH of 7.95. The reaction was carried out at 37 °C for 3 h in the dark. Following reduction, the sample was alkylated with IAM, and the mixture was subsequently purified using analytical HPLC and analyzed by MS and MS/MS analysis. The purified peptide was then subjected to chymotrypsin digestion in 20 mM Tris–HCl buffer (pH 7.95) at 37 °C for 3 h. After digestion, the sample was purified by RP-HPLC, and the resulting fragments were collected and further characterized by MS and MS/MS.

### BLAST Search of Transcriptome for Similar Sequences

To mine for stryphnine-like peptides, the sequence of stryphnine A was searched in the *S. fortis* transcriptome using a stand-alone tBLASTn with default settings. The cDNA fragments containing the hits were extracted and translated into an amino acid sequence using the Expasy translate tool (https://web.expasy.org/translate/). The precursor peptide sequences were aligned using the Clustal Omega online tool (https://www.ebi.ac.uk/jdispatcher/msa/clustalo). The SignalP 5.0 (https://services.healthtech.dtu.dk/services/SignalP-5.0/) was used for the identification of the signaling region in the precursor peptides.^51^

### Dot Blot Experiments

The dot blot experiments were performed according to previously published protocols^52^ with modifications: the primary antibody was rabbit antiphosphoserine/threonine polyclonal (Thermo Fisher), and the blocking solution was 3% bovine serum albumin solution in tris-buffered saline, 0.5% Tween 20, pH 7.4.

### Synthesis of Stryphnine A

Fmoc-Tyr­(*t*Bu)­Wang polystyrene (LL) with a substitution value of 0.29 mmol/g from CEM was employed in synthesis. Stryphnine A [Ser10-O–CO-Glu29] was synthesized on a 0.1 mmol scale using FMOC-SPPS. First residue Cys^31^ was double-coupled using 0.5 M HATU as a coupling agent and Dipea as base (4 eq of amino acid, 4 eq of HATU, and 6 eq of DIPEA). The rest of the amino acids were double-coupled using 0.5 M HBTU and DIPEA as base (4 eq of amino acid, 4 eq of HBTU, and 6 eq of DIPEA), until Cys17 was coupled. Thereafter, a pseudoproline dipeptide, Fmoc-Val-Cys­(psiDmp. Hbpro)­OH (Novabiochem) (2 eq amino acid, 2 eq 0.5 M HBTU, and 3 eq DIPEA) was introduced at the V15 and Cys16 positions (double coupled). The rest of the amino acids were double-coupled up to the last Cys^1^. The final Fmoc group was deprotected using 20% piperidine. The same protocol was followed for the phosphorylated peptide synthesis, except at position Ser10, intended for phosphorylation, Fmoc-Ser­(PO­(OBzl)­OH)–OH (Sigma) was coupled.

Following synthesis, the resin was washed with CH_2_Cl_2_, dried by N2 and cleaved. The cleaved peptide was purified by RP-HPLC. The pure peptide was subjected to 10 oxidation trials. Of these, condition 6, containing 25% isopropanol, 0.1 M NH_4_HCO_3_ (pH 8.5), 2 mM reduced glutathione, and 0.4 mM oxidized glutathione, was selected for large-scale oxidation. Following overnight oxidation, the isopropanol concentration was diluted to 20%, the sample was filtered and purified on a 3 mL/min column. Analytical RP-HPLC was run on the purified fractions, and the purest peptide samples, as determined by LC-UV, were freeze–dried and subjected to coelution analysis, NMR studies, and bioassays. The same folding conditions and subsequent purification steps were also applied to the phosphorylated peptide.

### Determination of the Presence of Dehydroalanine Unit

The presence of a dehydroalanine unit was determined as described by Chalker et al.^53^ In brief, 70 μL of each peptide after reduction and alkylation was added to 1.4 μL of benzyl mercaptan. The mixture was incubated at 37 °C for 30 min and directly analyzed using an Xevo G2-XS quadrupole time-of-flight (QToF) mass spectrometer coupled with a nano-acquity UPLC after required dilution.

### Stability of the Peptides under Different pH Conditions

The stability of peptides was determined at pH 1, 7, and 13 under the incubation at 70 °C as described by He et al.^54^ The buffer solutions at pH 1 (0.1 M HCl), pH 7 (0.1 M sodium phosphate (monobasic) and 0.1 M sodium phosphate (dibasic)), and pH 13 (0.1 M KCl and 0.1 M NaOH) were prepared freshly, and the pH was measured at 70 °C. The ionic strength of each buffer solution was maintained at 0.15 by the addition of the required amount of NaCl. Fifty microliter of stryphnine A [Ser10-O–CO-Glu29], stryphnine A, and stryphnine B [Ser11-O–CO-Glu30] in Milli-Q water (50 μM) was added to 1.5 mL glass autosampler vials containing 70 μL of each buffer solution. The vials were incubated at 70 °C in an oven, and 20 μL from each vial was withdrawn after 1 h and was stored in the freezer until further analysis. Each vial was diluted further using Milli-Q water and was directly analyzed using an Xevo G2-XS quadrupole time-of-flight (QToF) mass spectrometer coupled with a nano-Acquity UPLC. Each experiment was triplicated.

To confirm the identity of the resulting product, stryphnine A [Ser10-O–CO-Glu29] peptide was incubated in ammonium bicarbonate buffer (pH 8.5) at 70 °C for 2 h. After incubation, the sample was purified using analytical RP-HPLC, freeze–dried, and analyzed by NMR. Additionally, the sample was analyzed by mass spectrometry and compared with both native stryphnine A and synthetic stryphnine A.

### Stability Testing of Peptides in Human Serum

The stability of the peptides was determined at fixed time intervals: 0 h, 3 h, 6 h, 8 h, and 24 h according to an established protocol.^55^ Each stability test was triplicated. Eight microliter of stryphnine A [Ser10-O–CO-Glu29], stryphnine A, and stryphnine B [Ser11-O–CO-Glu30] (200 μM) in water was mixed with 80 μL of human serum (male type AB—Sigma-Aldrich, Germany) and was incubated at 37 °C with constant shaking. The proteins were denatured by adding 80 μL of 6 M urea to release the peptide into the solution at the required time interval. After incubation for 10 min at 5 °C, the serum proteins were precipitated by the addition of 80 μL of 20% trichloroacetic acid and were incubated again at 5 °C for another 10 min. The supernatant was obtained after centrifugation and analyzed using liquid chromatography coupled to a UV detector at 215 nm. The average peak area of a particular peptide at different time points was quantified as the % peptide remaining in serum. Phosphate buffered saline (PBS) was used as a control at time intervals 0 and 24 h.

### NMR Spectroscopy

NMR spectra were acquired on a Bruker Avance Neo 600 MHz equipped with a cryogenic TCI probe (CRPHe TR—^1^H&^19^F/^13^C/^15^N 5 mm-EZ. Approximately 2 mg of each of the lyophilized peptides was dissolved in 90% H_2_O/10% D_2_O (220 μL) at pH 5, and a series of ^1^H NMR, TOCSY, NOESY, and ^15^N HSQC spectra were acquired at 290 K or/and 298 K. These TOCSY and NOESY spectra were used for sequential chemical shift assignment and secondary αH chemical shift measurement. Generally, 4096 data points were collected in the F2 dimension and 256 (128 complex) points in F1 over 11 194 Hz. For amide temperature coefficient measurements, an additional set of TOCSY spectra was acquired on selected peptides by varying the temperature in intervals from 285 to 300 K. The peptides were then freeze–dried, dissolved in 220 μL of 100% D_2_O, and a series of ^1^H NMR and TOCSY spectra were acquired for hydrogen–deuterium exchange experiments. ^13^C HSQC and HMBC spectra were acquired on the same samples dissolved in 100% D_2_O. Chemical shift assignment of ^15^N NHSQC and ^13^C HSQC spectra was used for obtaining ϕ and ψ torsion angle constraints. ^31^P NMR was recorded on a Varian MR400 MHz spectrometer equipped with a OneNMR probe at 25 °C. Chemical shifts (ppm) are reported referenced to the internal signal of the residual protic solvent.

### Peptide Structure Determination

All NMR spectra were processed in Topspin 2.1 (Bruker), and chemical shift assignment was done in CCPNMR software.^56^ Resonance assignments of peptides were obtained by using sequential assignment strategies. Peptide backbone torsion angle constraints were obtained in the TALOS-N chemical shift analysis. H-bond restraints were identified by D_2_O experiments in conjunction with preliminary structures. Once the chemical shift assignments were identified from TOCSY spectra, all NOE connections were identified and converted into interproton distances using CYANA 3.98,^57^ and 50 random conformers were annealed in 8000 steps using torsion angle dynamics, of which 20 conformers with the lowest energies and the lowest residual restraint violations were selected to represent the solution structure of synthetic stryphnine A. The 3D structures were generated using MOLMOL, and structure qualities were validated using MolProbity.^58^

### Antibiofouling Testing

The biofouling activity of stryphnine A [Ser10-O–CO-Glu29] and stryphnine B [Ser11-O–CO-Glu30] was determined as described by Sjögren et al.^55,59^ Live cyprid larvae of the bay barnacle *A. improvisus* (Darwin, 1854) were obtained from the Tjärnö Marine Laboratory (University of Gothenburg, Sweden). Initially, 20 ± 2 cyprid larvae were incubated in 9.9 mL of filtered seawater (FSW) containing 100 μL of Milli-Q water (control) and 9.9 mL of FSW containing 100 μL of peptide in Milli-Q water (treatment). Both peptides were tested in three different concentrations: 2, 0.2, and 0.02 μM. The settlement of larvae was assessed after 3 days of incubation at room temperature. The experiment was carried out in triplicate.

### Neuraminidase (Sialidase Assay)

The neuraminidase assay was conducted using the Amplex^Tm^ Red neuraminidase assay kit (Thermo Fisher Scientific) in a 96-well microtiter plate format and the neuraminidase enzyme from *Clostridium perfringens* (Type V, lyophilized powder, 6 units, Sigma-Aldrich). A working solution was prepared to contain 100 μM Amplex Red, 0.2 U/mL horseradish peroxidase (HRP), 4 U/mL galactose oxidase, and 500 μg/mL fetuin in reaction buffer (prepared from a 5X reaction buffer containing 0.25 M Tris–HCl, pH 7.2, 5 mM CaCl_2_). Neuraminidase stock solution, at 1.5 U/mL was prepared in 1X Reaction Buffer. Additionally, the peptide stock solutions were prepared in Milli-Q at a concentration of 2 mg/mL. Different concentrations of the test peptides were preincubated with 2 μL of the neuraminidase stock solution in a total volume of 50 μL of 1X Reaction Buffer for 10 min at room temperature. The positive control contained 50 μL of 1X Reaction Buffer without neuraminidase enzyme, while the negative control consisted of 2 μL from the neuraminidase stock solution and 48 μL of 1X Reaction Buffer. After the 10 min preincubation time, 50 μL of the working solution was added to all wells (test compounds, positive control, and negative control), and the plate was incubated at 37 °C protected from light. The absorbance at 571 nm was measured at 30, 60, 90, and 120 min time intervals. The percentage of absorbance reduction was calculated relative to the negative control.

### Evaluation of Genotoxicity and Cytotoxicity

An in vitro version of the micronucleus (MN) assay^32^ was used to assess the genotoxicity of the three different peptides: stryphnine A [Ser10-O–CO-Glu29], stryphnine A, and stryphnine B [Ser11-O–CO-Glu30]. The same assay was also used to evaluate the cytotoxicity.

#### Cells

Chinese hamster ovary cells (CHO-K1, ATCC number CCL 61, USA) was maintained in a F-12K medium (Kaighn’s Modification of Ham’s F-12 Medium) (ATCC, USA), supplemented with 10% fetal bovine serum (FBS) (Fisher Scientific, USA) and 1% penicillin–streptomycin (Fisher Scientific, USA), in a humified atmosphere of 5% CO_2_ and a temperature of 37 °C. Cells were harvested for experiments in a 0.5% trypsin-EDTA solution (Fisher Scientific, USA). The cells used in the experiments were between passages 5 and 22.

#### MN-Assay

The in vitro micronucleus assay was conducted in accordance with the OECD guidelines.^60^ On the first day, the CHO-K1 cells were plated in a Corning 96 well Black Polystyrene Microplate (VWR, USA) with a total volume of 100 μL/well and then incubated overnight to ensure attachment of the cells. Approximately 8000 cells/well were seeded for the experiment (in triplicates for each individual exposure).

#### Exposure Conditions

The cells were exposed to varying concentrations (1–40 μM) of the three different peptides, vehicle (negative control), or 14 μM of a known clastogen and aneugen, mitomycin C (MMC, the positive control; Sigma-Aldrich, USA), for 3 h. Following exposure, the treatment solutions were discarded, and the cells were immediately washed with serum-free medium. To ensure analysis of only those cells that completed mitosis during or after the exposure, cytochalasin B (CytoB, Sigma-Aldrich, USA), the actin polymerization inhibitor, was added (100 μL of 3 μg/mL CytoB in 5% FBS medium per well). The cells were then incubated for another 45 h. After the cytoB solution was removed, the cells were subjected to another 30 min staining process using a solution containing 1 mM of the fluorescent dye Mitotracker Orange (Fisher Scientific, USA) and 2 mM Hoechst 33342 (Fisher Scientific, USA) in 5% FBS medium. Following staining, the cells were washed with phosphate-buffered saline (PBS, Fisher Scientific, USA) and then fixed with 4% paraformaldehyde (100 μL/well; Sigma-Aldrich, USA) for 10 min at room temperature, shielded from light. In the final step, the cells were washed twice with PBS and stored in the dark until the day of the image analysis.

#### Image Analysis

The image analysis was conducted using an ImageXpress micro XLS instrument (Molecular Devices, USA) coupled with MetaXpress software. For the genotoxicity analysis, at least 2000 BNC were scored per concentration, including the negative vehicle control, to determine the frequency of micronuclei (% MN frequency) in the BNC. The cells and the micronuclei were detected on the basis of their size and intensity criteria. Moreover, the maximum distance of nuclei in polynucleated cells was set to characterize cells as mononucleated, binucleated, or multinucleated. Formation of micronuclei in BNC indicates chromosomal damage that has persisted into daughter cells. Introduction of cytochalasin B, which inhibits, thus prevents the separation of daughter cells in mitosis, facilitating identification of BNC with micronuclei. The genotoxicity of the tested substances is quantified as the percentage of BNC with micronuclei (% BNC with MN). The cytotoxicity was calculated using the cytokinesis-block proliferation index (CBPI), which indicates the average number of nuclei per cell. The cytotoxicity for a given treatment is evaluated by comparing the CBPI of treated cultures to that of control cultures, expressed as a percentage.

Statistical analysis was conducted on data pooled from three independent experiments, using triplicates for each individual exposure (*n* = 9). A two-tailed *t*-test with independent samples and equal variance was performed to identify statistically significant differences (*p* < 0.001) by comparing the mean values ± standard error of means (SEM) between the treated cultures and the negative vehicle controls.

### Microdilution Assay

The antimicrobial activity of the peptides was determined against *S. aureus* (ATCC 29213), *E. coli* (ATCC 25922), and *C. albicans* (ATCC 90028) in triplicate. Bacterial strains were obtained from the Department of Clinical Bacteriology, Lund University Hospital, Lund, Sweden. An established protocol^35^ was followed. Microbial strains were first cultured at 37 °C to mid-logarithmic phase on 3% tryptic soy broth (TSB, Merck KGaA, Germany). Bacterial cells were washed twice by centrifugation and resuspension in Tris buffer (10 mM, pH 7.8 at RT) to 100,000 CFU/mL (measured at OD600). Fifty microliter of tris buffer was added to each well, and 50 μL of 200 μM pure peptides (stryphnine A [Ser10-O–CO-Glu29], stryphnine A, and stryphnine B [Ser11-O–CO-Glu30]) in Milli-Q water was added only to the first well. The two-step serial dilution was carried out, followed by the addition of 50 μL of bacterial suspension. After 5 h incubation at 37 °C, each well was administered 5 μL of 20% TSB (low temp, sterilized at 100 °C), and the plates were reincubated 6–9 h depending on the growth rate of each bacterial strain used. The potency of the antimicrobial activity was recorded by visible inspection of the wells exhibiting a total growth inhibition. A volume of 100 μL of tris buffer was used as the negative control, and the human antimicrobial peptide LL-37 (5 μM) was used as the positive control.

## Supplementary Material



## Data Availability

The structure of stryphnine A and related chemical shifts have been deposited in the Protein Data Bank (RCSB PDB) and the Biomagnetic Resonance Bank (BMRB) with accession codes 8VAY and 31135, respectively. The underlying raw NMR FID/serial files, pulse sequences, and acquisition parameters are archived in Zenodo and can be accessed at https://doi.org/10.5281/zenodo.20126511

## Notes

Peptide-like MW observed in the 60% CH_3_CN in water fraction of *S. fortis*, MS/MS fragments for the enzyme cleaved fragments, nucleotide sequences containing different stryphnines found in the *S. fortis* transcriptome, oxidation trials, amide coefficients, LCMS analysis of isolated stryphnines, treatment of stryphnine A [Ser10-O–CO-Glu29] with benzylmercaptan, experiments to rule out phosphorylation in stryphnine A, confirmation of the correct folding of synthetic stryphnine A, stability experiments of stryphnines, comparison of the fingerprint region of the TOCSY and NOESY spectra for native and synthetic stryphnine A, comparison of the fingerprint region of the TOCSY and NOESY spectra of stryphnine A [Ser10-O–CO-Glu29] and stryphnine B [Ser11-O–CO-Glu30], and neuraminidase (sialidase) inhibitory activity of the peptides (PDF)

## References

[1] Parsley N. C., Williams O. L., Hicks L. M. (2020). Exploring the Diversity of Cysteine-Rich
Natural Product Peptides via MS/MS Fingerprint Ions. J. Am. Soc. Mass Spectrom..

[2] Shen Y., Xu L., Huang J., Serra A., Yang H., Tam J. P. (2019). Potentides:
New Cysteine-Rich Peptides with Unusual Disulfide Connectivity from *Potentilla anserina*. ChemBioChem.

[3] Ho T. N. T., Turner A., Pham S. H., Nguyen H. T., Nguyen L. T. T., Nguyen L. T., Dang T. T. (2023). Cysteine-Rich
Peptides: From Bioactivity
to Bioinsecticide Applications. Toxicon.

[4] Wang C. K., Craik D. J. (2018). Designing Macrocyclic
Disulfide-Rich Peptides for Biotechnological
Applications Perspective. Nat. Chem. Biol..

[5] Meng X., Xu C., Fan S., Dong M., Zhuang J., Duan Z., Zhao Y., Wu C. (2023). Selection and Evolution of Disulfide-Rich
Peptides via Cellular Protein Quality Control. Chem. Sci..

[6] González-Castro R., Gómez-Lim M. A., Plisson F. (2021). Cysteine-Rich Peptides: Hyperstable
Scaffolds for Protein Engineering. ChemBioChem.

[7] Correnti C. E., Gewe M. M., Mehlin C., Bandaranayake A. D., Johnsen W. A., Rupert P. B., Brusniak M. Y., Clarke M., Burke S. E., De Van Der Schueren W., Pilat K., Turnbaugh S. M., May D., Watson A., Chan M. K., Bahl C. D., Olson J. M., Strong R. K. (2018). Screening,
Large-Scale Production and Structure-Based
Classification of Cystine-Dense Peptides. Nat.
Struct. Mol. Biol..

[8] Han, B. ; Hong, L. ; Gu, B. ; Sun, Y. ; Wang, J. Natural Products from Sponges. In Symbiotic Microbiomes of Coral Reefs Sponges and Corals; Springer: Dordrecht, 2019; pp 329–463.

[9] Kosgahakumbura L., Gamage J., Hettiarachchi C. M., Cárdenas P., Gunasekera S. (2025). Ribosomally Synthesised and Post-Translationally
Modified
Peptides (RiPPs) from Marine Demosponges and Their Microsymbionts. Aust. J. Chem..

[10] Carstens B. B., Rosengren K. J., Gunasekera S., Schempp S., Bohlin L., Dahlström M., Clark R. J., Göransson U. (2015). Isolation,
Characterization, and Synthesis of the Barrettides: Disulfide-Containing
Peptides from the Marine Sponge *Geodia barretti*. J. Nat. Prod..

[11] Steffen K., Laborde Q., Gunasekera S., Payne C. D., Rosengren K. J., Riesgo A., Göransson U., Cárdenas P. (2021). Barrettides:
A Peptide Family Specifically Produced by the Deep-Sea Sponge *Geodia barretti*. J. Nat. Prod..

[12] Steffen K., Proux-Wéra E., Soler L., Churcher A., Sundh J., Cárdenas P. (2023). Whole Genome
Sequence of the Deep-Sea Sponge *Geodia barretti* (Metazoa,
Porifera, Demospongiae). G3:Genes, Genomes,
Genet..

[13] Krumpe L. R. H., Wilson B. A. P., Marchand C., Sunassee S. N., Bermingham A., Wang W., Price E., Guszczynski T., Kelley J. A., Gustafson K. R., Pommier Y., Rosengren K. J., Schroeder C. I., O’Keefe B. R. (2020). Recifin A, Initial Example of the
Tyr-Lock Peptide Structural Family, Is a Selective Allosteric Inhibitor
of Tyrosyl-DNA Phosphodiesterase. J. Am. Chem.
Soc..

[14] Williams D. E., Austin P., Diaz-marrero A. R., Soest R. V., Matainaho T., Roskelley C. D., Roberge M., Andersen R. J. (2005). Neopetrosiamides,
Peptides from the Marine Sponge *Neopetrosia* sp. That
Inhibit Amoeboid Invasion by Human Tumor Cells. Org. Lett..

[15] Matsunaga S., Jimbo M., Gill M. B., Lash-Van Wyhe L. L., Murata M., Nonomura K., Swanson G. T., Sakai R. (2011). Isolation,
Amino Acid Sequence and Biological Activities of Novel Long-Chain
Polyamine-Associated Peptide Toxins from the Sponge *Axinyssa
aculeata*. ChemBioChem.

[16] Takada K., Hamada T., Hirota H., Nakao Y., Matsunaga S., van Soest R. W. M., Fusetani N. (2006). Asteropine A, a Sialidase-Inhibiting
Conotoxin-like Peptide from the Marine Sponge *Asteropus simplex*. Chem. Biol..

[17] Li H., Bowling J. J., Fronczek F. R., Hong J., Jabba S. V., Murray T. F., Ha N. C., Hamann M. T., Jung J. H. (2013). Asteropsin
A: An Unusual Cystine-Crosslinked Peptide from Porifera Enhances Neuronal
Ca^2+^ Influx. Biochim. Biophys. Acta,
Gen. Subj..

[18] Li H., Bowling J. J., Su M., Hong J., Lee B. J., Hamann M. T., Jung J. H. (2014). Asteropsins,
B-D, Sponge-Derived
Knottins with Potential Utility as a Novel Scaffold for Oral Peptide
Drugs. Biochim. Biophys. Acta, Gen. Subj..

[19] Su M., Li H., Wang H., Kim E. L., Kim H. S., Kim E. H., Lee J., Jung J. H. (2016). Stable and Biocompatible Cystine Knot Peptides from
the Marine Sponge *Asteropus* sp. Bioorg. Med. Chem..

[20] Li H., Su M., Hamann M. T., Bowling J. J., Kim H. S., Jung J. H. (2014). Solution
Structure of a Sponge-Derived Cystine Knot Peptide and Its Notable
Stability. J. Nat. Prod..

[21] Cárdenas P., Xavier J. R., Reveillaud J., Schander C., Rapp H. T. (2011). Molecular
Phylogeny of the Astrophorida (Porifera, Demospongiae^p^)
Reveals an Unexpected High Level of Spicule Homoplasy. PLoS One.

[22] Cárdenas P., Rapp H. T. (2015). Demosponges from the Northern Mid-Atlantic Ridge Shed
More Light on the Diversity and Biogeography of North Atlantic Deep-Sea
Sponges. J. Mar. Biol. Assoc. U. K..

[23] Moodie L. W. K., Žužek M. C., Frangež R., Andersen J. H., Hansen E., Olsen E. K., Cergolj M., Sepčić K., Hansen K., Svenson J. (2016). Synthetic Analogs of
Stryphnusin Isolated from the Marine Sponge: *Stryphnus fortis* Inhibit Acetylcholinesterase with No Effect on Muscle Function or
Neuromuscular Transmission. Org. Biomol. Chem..

[24] Hanssen K., Cervin G., Trepos R., Petitbois J., Haug T., Hansen E., Andersen J. H., Pavia H., Hellio C., Svenson J. (2014). The Bromotyrosine Derivative
Ianthelline
Isolated from the Arctic Marine Sponge *Stryphnus fortis* Inhibits Marine Micro- and Macrobiofouling. Mar. Biotechnol..

[25] Hanssen K., Andersen J. H., Stiberg T., Engh R. A., Svenson J., Genevière A. M., Hansen E. (2012). Antitumoral and Mechanistic
Studies
of Ianthelline Isolated from the Arctic Sponge *Stryphnus fortis*. Anticancer Res..

[26] Cárdenas P. (2016). Who Produces
Ianthelline? The Arctic Sponge *Stryphnus fortis* or
Its Sponge Epibiont *Hexadella dedritifera*: A Probable
Case of Sponge-Sponge Contamination. J. Chem.
Ecol..

[27] Plese B., Kenny N. J., Rossi M. E., Cárdenas P., Schuster A., Taboada S., Koutsouveli V., Riesgo A. (2021). Mitochondrial Evolution in the Demospongiae (Porifera):
Phylogeny, Divergence Time, and Genome Biology. Mol. Phylogenet. Evol..

[28] McIntosh J. A., Donia M. S., Schmidt E. W. (2009). Ribosomal
Peptide Natural Products:
Bridging the Ribosomal and Nonribosomal Worlds. Nat. Prod. Rep..

[29] Geoghegan K. F., Hoth L. R., Tan D. H., Borzilleri K. . A., Withka J. M., Boyd J. . G. (2002). Cyclization of N-Terminal S-Carbamoylmethylcysteine
Causing Loss of 17 Da from Peptides and Extra Peaks in Peptide Maps. J. Proteome Res..

[30] Tinette S., Feyereisen R., Robichon A. (2007). Approach to Systematic Analysis of
Serine/Threonine Phosphoproteome Using Beta Elimination and Subsequent
Side Effects: Intramolecular Linkage and/or Racemisation. J. Cell. Biochem..

[31] Conibear A. C., Rosengren K. J., Becker C. F. W., Kaehlig H. (2019). Random Coil Shifts
of Posttranslationally Modified Amino Acids. J. Biomol. NMR.

[32] Fenech M. (2000). The in Vitro
Micronucleus Technique. Mutat. Res., Fundam.
Mol. Mech. Mutagen..

[33] Fenech M. (2007). Cytokinesis-Block
Micronucleus Cytome Assay. Nat. Protoc..

[34] Vitali, A. Antimicrobial Peptides Derived from Marine Sponges. Am. J. Clin. Microbiol. Antimicrob. 2018, 1(1) 1–11.

[35] Strömstedt A.
A., Park S., Burman R., Göransson U. (2017). Bactericidal
Activity of Cyclotides Where Phosphatidylethanolamine-Lipid Selectivity
Determines Antimicrobial Spectra. Biochim. Biophys.
Acta, Biomembr..

[36] Andavan G. S., Lemmens-Gruber R. (2010). Cyclodepsipeptides from Marine Sponges:
Natural Agents
For Drug Research. Mar. Drugs.

[37] Zeng M., Tao J., Xu S., Bai X., Zhang H. (2023). Marine Organisms as
a Prolific Source of Bioactive Depsipeptides. Mar. Drugs.

[38] IUPAC . Depsipeptides. In IUPAC Compendium of Chemical Terminology; International Union of Pure and Applied Chemistry, 2014.

[39] D’Auria M. V., Zampella A., Paloma L. G., Minale L., Debitus C., Roussakis C., Le Bert V. (1996). Callipeltins B and C; Bioactive Peptides
from a Marine Lithistida Sponge *Callipelta* sp. Tetrahedron.

[40] Ratnayake A. S., Bugni T. S., Feng X., Harper M. K., Skalicky J. J., Mohammed K. A., Andjelic C. D., Barrows L. R., Ireland C. (2006). M. Theopapuamide,
a Cyclic Depsipeptide from a Papua New Guinea Lithistid Sponge *60*. J. Nat. Prod..

[41] Robinson S. J., Morinaka B. I., Amagata T., Tenney K., Bray W. M., Gassner N. C., Lokey R. S., Crews P. (2010). New Structures and
Bioactivity Properties of Jasplakinolide (Jaspamide) Analogues from
Marine Sponges. J. Med. Chem..

[42] Sato K., Horibe K., Amano K. I., Mitusi-Saito M., Hori M., Matsunaga S., Fusetani N., Ozaki H., Karaki H. (2001). Membrane Permeabilization
Induced by Discodermin A,
a Novel Marine Bioactive Peptide. Toxicon.

[43] Amaral S. C. D., Monteiro P. R., Neto J. d. S. P., Serra G. M., Gonçalves E. C., Xavier L. P., Santos A. V. (2021). Current
Knowledge on Microviridin
from Cyanobacteria. Mar. Drugs.

[44] Weckesser J., Martin C., Jakobi C. C. (1996). Cyanopeptolins,
Depsipeptides from
Cyanobacteria. Syst. Appl. Microbiol..

[45] Eggen M., Georg G. I. (2002). The Cryptophycins:
Their Synthesis and Anticancer Activity. Med.
Res. Rev..

[46] Lee H., Choi M., Park J.-U., Roh H., Kim S. (2020). Genome Mining
Reveals High Topological Diversity of Ω-Ester- Containing Peptides
and Divergent Evolution of ATP-Grasp Macrocyclases. J. Am. Chem. Soc..

[47] Choi B., Link A. J. (2023). Discovery, Function, and Engineering of Graspetides. Trends Chem..

[48] Kwon H., Squire C. J., Young P. G., Baker E. N. (2014). Autocatalytically
Generated Thr-Gln Ester Bond Cross-Links Stabilize the Repetitive
Ig-Domain Shaft of a Bacterial Cell Surface Adhesin. Proc. Natl. Acad. Sci. U.S.A..

[49] de Voogd, N. J. ; Alvarez, B. ; Boury-Esnault, N. ; Cárdenas, P. ; Díaz, M.-C. ; Dohrmann, M. ; Downey, R. ; Goodwin, C. ; Hajdu, E. ; Hooper, J. N. A. ; Kelly, M. ; Klautau, M. ; Lim, S. C. ; Manconi, R. ; Morrow, C. ; Pinheiro, U. ; Pisera, A. B. ; Ríos, P. ; Schönberg, C. ; Turner, T. ; Vacelet, J. ; van Soest, R. W. M. ; Xavier, J. World Porifera Database (accessed Apr 05, 2024). https://www.marinespecies.org/porifera.

[50] Rice P., Longden L., Bleasby A. (2000). EMBOSS: The
European Molecular Biology
Open Software Suite. Trends Genet..

[51] Almagro
Armenteros J. J., Tsirigos K. D., Sønderby C. K., Petersen T. N., Winther O., Brunak S., von Heijne G., Nielsen H. (2019). SignalP 5.0 Improves Signal Peptide Predictions Using
Deep Neural Networks. Nat. Biotechnol..

[52] Slazak B., Kapusta M., Malik S., Bohdanowicz J., Kuta E., Malec P., Göransson U. (2016). Immunolocalization
of Cyclotides in Plant Cells, Tissues and Organ Supports Their Role
in Host Defense. Planta.

[53] Chalker J. M., Lercher L., Rose N. R., Schofield C. J., Davis B. G. (2012). Conversion of Cysteine into Dehydroalanine Enables
Access to Synthetic Histones Bearing Diverse Post-Translational Modifications. Angew. Chem., Int. Ed..

[54] He H. T., Gürsoy R. N., Kupczyk-subotkowska L., Tian J., Williams T., Siahaan T. J. (2006). Synthesis and Chemical Stability of a Disulfide Bond
in a Model Cyclic Pentapeptide: Cyclo­(1,4)-Cys-Gly-Phe-Cys-Gly-OH. J. Pharm. Sci..

[55] Gunasekera S., Foley F. M., Clark R. J., Sando L., Fabri L. J., Craik D. J., Daly N. L. (2008). Engineering Stabilized Vascular Endothelial
Growth Factor-A Antagonists: Synthesis, Structural Characterization,
and Bioactivity of Grafted Analogues of Cyclotides. J. Med. Chem..

[56] Vranken W. F., Boucher W., Stevens T. J., Fogh R. H., Pajon A., Llinas M., Ulrich E. L., Markley J. L., Ionides J., Laue E. D. (2005). The CCPN Data Model for NMR Spectroscopy: Development
of a Software Pipeline. Proteins:Struct., Funct.,
Bioinf..

[57] Güntert, P. Automated NMR Structure Calculation with CYANA. In Protein NMR Techniques; Humana Press, 2004; Vol. 278, pp 353–378. 10.1385/1-59259-809-9:353 15318003

[58] Chen V. B., Arendall W. B., Headd J. J., Keedy D. A., Immormino R. M., Kapral G. J., Murray L. W., Richardson J. S., Richardson D. C. (2010). MolProbity: All-Atom Structure Validation for Macromolecular
Crystallography. Acta Crystallogr., Sect. D:Biol.
Crystallogr..

[59] Sjögren M., Johnson A., Hedner E., Dahlstro M., Shirani H., Bergman J., Jonsson P. R., Bohlin L. (2006). Antifouling Activity
of Synthesized Peptide Analogs of the Sponge Metabolite Barettin. Peptides.

[60] OECD . Test Guideline No. 487: In Vitro Mammalian Cell Micronucleus Test. In OECD Guidelines for the Testing of Chemicals, Section 4; OECD Publishing: Paris, 2023; pp 1–29.

