# Supplementary material for: Discovery of Ribosomally Synthesized and Post-translationally Modified Knottins from the Deep-Sea Sponge Stryphnus fortis
Source: J Nat Prod. 2026 Jun 9;89(7):2118–32. doi: 10.1021/acs.jnatprod.6c00501 (PMC13418204; doi:10.1021/acs.jnatprod.6c00501)
Supplement: Supplementary file 1 [file np6c00501_si_001.pdf]

**Supplementary Information**

# Discovery of ribosomally-synthesized and post-translationally modified knottins from the deep-sea sponge *Stryphnus fortis*

Lakmini N. Kosgahakumbura[a,b], Blazej Slazak[a,c], Norelle L. Daly[d], Rein Fadoul[e], Samia Mohamed[a], Erik Jacobsson[a], Ruisheng Xiong[f], Björn Hellman[e], Ulf Göransson[a], Chamari M. Hettiarachchi [b], Paco Cárdenas [a,g], Sunithi Gunasekera [a,*]

[a] Pharmacognosy, Department of Pharmaceutical Biosciences, Biomedical Center, Uppsala University, Box 591, SE-751 24, Uppsala, Sweden

[b] Department of Chemistry, University of Colombo, Colombo 00300, Sri Lanka

[c] W. Szafer Institute of Botany, Polish Academy of Science, 46 Lubicz St., 31-512 Kracow, Poland

[d] Australian Institute of Tropical Health and Medicine, James Cook University, Cairns, Australia

[e] Drug Safety and Toxicology, Department of Pharmaceutical Biosciences, Biomedical Center, SE-751 24 Uppsala, Sweden

[f] SciLifeLab, Department of Chemistry for Life Sciences, Uppsala University, Box 576, SE-75123, Uppsala, Sweden

[g] Museum of Evolution, Uppsala University, Norbyvägen 16, SE-752 36 Uppsala, Sweden

**Table of contents**


| Item | Page number |
|---|---|
| **Table S1**: The peptide-like molecular weights (MW) observed in the 60% $CH_3CN$ in water fraction of *S. fortis* | S-3 |
| **Table S2**. Table with MS/MS fragments for the enzyme cleaved fragments | S-4 |
| **Table S3**: Nucleotide sequences containing different stryphnines found in the *S. fortis* transcriptome | S-5 |
| **Table S4.** Oxidation trials | S-7 |
| **Table S5**: Amide co-efficients | S-8 |
| **Fig. S1.** LCMS analysis of stryphnines | S-9 |
| **Fig. S2**. Treatment of stryphnine A [Ser10-O-CO-Glu29] with benzylmercaptan. | S-10 |
| **Fig. S3:** Experiments conducted to rule out phosphorylation in stryphnines | S-11 |
| **Fig. S4**. Confirmation of the correct folding of synthetic stryphnine A | S-12 |
| **Fig. S5**. Comparison of the fingerprint region of the TOCSY and NOESY spectra for native and synthetic stryphnine A | S-13 |
| **Fig. S6**. Comparison of the fingerprint region of the TOCSY and NOESY spectra of stryphnine A [Ser10-O-CO-Glu29] and stryphnine B [Ser11-O-CO-Glu30] | S-14 |
| **Fig. S7.** Chemical, thermal, and human serum stabilities of stryphnines subjected to different pH treatments at elevated temperature (70 °C). | S-15 |
| **Fig. S8**: Neuraminidase (Sialidase) inhibitory activity of the peptides | S-16 |

Table S1: The most abundant peptide-like molecular weights (MW, calculated from m/z 3+ or 4+ ions) from *S. fortis* observed in the fraction of the extract eluted from C18 SPE with 60% $CH_3CN$ in water. Possible pairs of peptides with and without ester-modification (-18 Da difference) are marked with red and blue respectively.

| **Monoisotopic MW** |
|---|
| *3330.39 |
| *3348.39 |
| *3516.47 |
| 3077.24 |
| 3091.22 |
| 3092.23 |
| 3109.23 |
| 3293.28 |
| 3404.36 |
| 3498.49 |
| 3503.51 |
| 3607.48 |
| 3669.61 |
| 3720.59 |
| 3774.49 |
| 3784.61 |
| 3836.57 |
| 3850.72 |
| 3895.61 |
| 3913.65 |
| 3918.73 |
| 3945.70 |
| 4092.73 |
| 4165.75 |
| 4183.77 |
| 4278.86 |
| 4296.86 |
| 4605.98 |
| 4623.28 |
| 4775.36 |

* The monoisotopic parent masses of 3330.39 Da, 3348.39 Da, and 3516.47 Da were later confirmed as stryphnine A [Ser10-O-CO-Glu29], stryphnine A, and stryphnine B [Ser11-O-CO-Glu30], respectively.

Table S2: Table with MS/MS fragments for the enzyme cleaved fragments

| Peptide | Enzyme | Fragment | Expected mass (m/z) | Observed mass/ m/z |
|---|---|---|---|---|
| stryphnine A | chymotrypsin | CLPKGTPCPSTHPY* | $[M+2]^{2+}$ = 807.873 | $[M+2]^{2+}$ = 799.372 |
| | | GVPGAHEICY | $[M+1]^{1+}$ = 1102.498 | $[M+1]^{1+}$ = 1102.574 |
| | | VCCSGICY | $[M+1]^{1+}$ =1018.379 | $[M+1]^{1+}$ = 1018.379 |
| | trypsin | CLPK | $[M+1]^{1+}$ =517.280 | $[M+1]^{1+}$ = 517.280 |
| | | GTPCPSTHPYVCCSG ICYGVPGAHEICY | $[M+1]^{1+}$ =1067.218 | $[M+1]^{1+}$ =1061.176 |
| stryphnine A [Ser10-O-CO-Glu29] | chymotrypsin | CLPKGTPCPSTHPY* | $[M+2+]^{2+}$ = 798.868 | $[M+2]^{2+}$ = 790.367 |
| | | GVPGAHEICY | $[M+1]^{1+}$ = 1102.498 | $[M+1]^{1+}$ = 1102.574 |
| | | VCCSGICY | $[M+1]^{1+}$ =1018.379 | $[M+1]^{1+}$ = 1018.379 |
| | trypsin | CLPK | $[M+1]^{1+}$ =517.280 | $[M+1]^{1+}$ = 517.280 |
| | | GTPCPSTHPYVCCSG ICYGVPGAHEICY | $[M+1]^{1+}$ =1061.108 | $[M+1]^{1+}$ =1061.176 |
| stryphnine B [Ser11-O-CO-Glu30] | chymotrypsin | W# | $[M+1]^{1+}$ = 204.10 | - |
| | | CLPKGTPCPSTHPY* | $[M+2]^{2+}$ = 798.868 | $[M+2]^{2+}$ = 790.367 |
| | | GVPGAHEICY | $[M+1]^{1+}$ = 1102.498 | $[M+1]^{1+}$ = 1102.574 |
| | | VCCSGICY | $[M+1]^{1+}$ =1018.379 | $[M+1]^{1+}$ = 1018.379 |
| | trypsin | WCLPK | $[M+1]^{1+}$ =703.359 | $[M+1]^{1+}$ = 703.360 |
| | | GTPCPSTHPYVCCSG ICYGVPGAHEICY | $[M+1]^{1+}$ =1061.108 | $[M+1]^{1+}$ =1061.176 |

*A reduction of the observed mass by 17 Da compared to the expected mass due to N-terminal S-carbamoylmethylcysteine cyclization

#Cleaved W, a single amino acid was not detected in LCMS likely due to its polar nature.

Table S3. Nucleotide sequences containing different stryphnines found in the *S. fortis* transcriptome

| Sequence ID | Nucleotide sequence |
|---|---|
| TRINITY_DN106_c0_g1_i6 | TAAGCGGAAGTATAGAGTCGAGACATTCATTATCAAC<br>AGTGAAGCAACCGAAAGTTTACAAGTGAAGCAACTCT<br>CCTCAAACCAGAAGCAAAATGCGTGCTCTTCTACTCC<br>TCTGCACTGTGGGTATAGTGATGGCCTCTGTCATCGAT<br>CTCACATCGGCTCGTTACGTGCCACAAGGATACGAGG<br>CTAGAGCTCCAGCTCGCTACATGGAAGCCGATGAAGG<br>GTGCCTTCCAAAAGGAACTCCATGTCCCTCTACTCACC<br>CTTATGTATGCTGCTCTGGAATTTGCTATGGCGTCCCA<br>GGGGCGCATGAGATCTGTTATTAAAGATTGAAGATTG<br>GAAAGAAACCCTTTGAAACTGCATAAAATAGTTACCA<br>GTATAAAATATTTCATTTGCTTTATGACGTCAC |
| TRINITY_DN106_c0_g1_i10 | TAAGCGGAAGTATAGAGTCGAGACATTCATTATCAAC<br>AGTGAAGCAACCGAAAGTTTACAAGTGAAGCAACTCT<br>CCTCAAACCAGAAGCAAAATGCGTGCTCTTCTACTCC<br>TCTGCACTGTGGGTATAGTGATGGCCTCTGTCATCGAT<br>CTCACATCGGCTCATTACGTGCCACAAGGATACAATG<br>CTAGAGCTCCAGCTCGCTCCATGCAAGAACCAGAAGC<br>AGAACCAGAAGCAGAAGCAGAACCAGAACGAAAACC<br>AGAACGAAAACCAGAACCAGAACGAGAACCAACACC<br>AGAACCAACACCAGAACCATGGTGCTTTCCAAAAGGA<br>ACTCCATGTCCCTCTACTCACCCTTATGTATGCTGCTC<br>TGGAATTTGCTATGGCGTCCCAGGGGCGCATGAGATC<br>TGTTATTAAAGATTGAAGATTGGAAAGA |
| TRINITY_DN106_c0_g1_i12 | CCGATCCACGGTTAAGTAACCCGAAGTTTAAAAGTGA<br>AGCAAGTCTCCTCAAACCAGAAGCAAAATGCGTGCTC<br>TTCTACTCCTCTGCACTGTGGGTATAGTGATGGCCTCT<br>GTCATTCATCTCACATCGGCTGCTTACATGCCGCAAGG<br>ATACAATGCTAGAGCTCCAGCTCGCTCCATGGAAGAA<br>AATGCTGATGAATGGTGCATTCCAAGTGGATGGCCGT<br>GTTCCTCTGATTGGCTAATTAAATGCTGCTCTGGACTC<br>TGCTATCCGGCGGTCCCGTTTGGTATCTGTTAAAGATT<br>GAAGATTGGAAAGAAACCCTTTGAAACTGCATAAAAT<br>AGTTACCAGTATAAAATATTTCATTTGCTTTATGACGT<br>CAC |
| TRINITY_DN106_c0_g1_i11 | GATTCATTATCAACGGTGAAGCAACCGAAAGTTTAAA<br>GTGAAACAAGTCTCCTCAAACCAGAAGGAAAATGCGT<br>GCTCTTCTACTCCTCTGCACTGTGGGTATAGTGATGGC<br>CTCTGTCATCAATCTCACATCGGCTAGTTACATGCCAC<br>AAGGATACAATGCTAGAGCTCCAGCTCGCTCAATGGA<br>AGCCGATATACCATGGTGCCTTCCAAAGGGATGGATA<br>TGTACACCTGATTGGGGATATAAATGCTGCTCTGGAG |

| | |
|---|---|
| | TATGCAGGTCTCTGCTCCCATATAGTAGATGTGATTAA AGATTGGAAGATTGGAAAGAAACCCTTTGAAACTGCA TAAAATAGTTACCAGTATAAAATATTTCATTTGCTTTA TGACGTCAC |
| TRINITY_DN106_c0_g1_i8 | TAAGCGGAAGTATAGAGTCGAGACATTCATTATCAAC AGTGAAGCAACCGAAAGTTTACAAGTGAAGCAACTCT CCTCAAACCAGAAGCAAAATGCGTGCTCTTCTACTCC TCTGCACTGTGGGTATAGTGATGGCCTCTGTCATTCAT CTCACATCGGCTGCTTACATGCCGCAAGGATACAATG CTAGAGCTCCAGCTCGCTCCATGCAAGAAAATTCTGA TGAATGGTGCATTCTAAGTGGATGGCCGTGTTCCTCTG ATTGGCTAATTAAATGCTGCTCTGGACTCTGCTATCCG GCGCTCCCGTTTGGTATCTGTGCTTAAAGATTGCA |
| TRINITY_DN106_c0_g1_i2 | CCGATCCACGGTTAAGTAACCCGAAGTTTAAAAGTGA AGCAAGTCTCCTCAAACCAGAAGCAAAATGCGTGCTC TTCTACTCCTCTGCACTGTGGGTATAGTGATGGCCTCT GTCATTGATCTCACATCGGCTCGTTACATGCCACAAG GATACAATGCTAGGGCTCCAGCTCGCTTCATGGAAGA AGAAAATACCGATCAAAATGTGTGCCTTCCAAGTGGA TATGCGTGTTCCCCTAATATTCCTTGGCCTTGCTGCTC TACATTGTGCTATCCAGGGTACCCATTTGGTACCTGTG GTTAAAGATTGGA |
| TRINITY_DN35634_c0_g4_i2 | ACAAGGATACAATGCTAGAGCTCCAGCTCGCTCCATG GAAGAAAATGCCGATGAATGGTGCATTCCACCTGGAT ATTCGTGTTTACCTGCACTGCCTTGGGAATGCTGCTCT ACTGTGTGCTATCCGGTCCCGTGGCCGACCACGTCTG GCGGGATCTGTGCTTAAAGATTGGAAGATTGGAAAGA AACCCTTTGAAACAGTTACCTGTACATAATATTTCATT TGCTATAATAGATGTCACGGATGCTACGCATGTGTGA AATGGAGCTGTCGTTTACTTGAGAAAAATGTAGTTTG CGGACC |
| TRINITY_DN35634_c0_g4_i1 | ACAAGGATACAATGCTAGAGCTCCAGCTCGCTCCATG GAAGAAAATGCCGATGAATGGTGCATTCCACCTGGAT ATTCGTGTTTACCTGCACTGCCTTGGGAATGCTGCTCT ACTGTGTGCTATCCGGTCCCGTGGCCGACCACGTCTG GCGGGATCTGTGCTTAAAGATGGAAATAAACCCTTGA AACTGCATAATTATATAGTTACCTGTACATAATATTTC ATTTGCTATAATAGATGTCACGGATGCTACGCATGTGT GAAATGGAGCTGTCGTTTACTTGAGAAAAATGTAGTT TGCGGACC |
| TRINITY_DN21129_c0_g1_i3 | TGTGGGTGTAGTGATGGCCTCTGTCATTGATCTCACAT CGGCTCGTTACATGCCACAAGGATACAATGCTAGGGC TCCAGCTCACTCCATGGAAGAAGAAAATGCCGATCAA GAATTGTGCTTTCCAAGTGGATATCCGTGTTCCCCTTA TTGGATAATTCACTGCTGCTCTGCATTGTGCTATGGAG GGTACCCATATGGTACCTGTGCTTAAAGATTGGGA |

Table S4: Oxidation trials

| Condition # | Peptide volume in µl (2 mg/ml) | 0.1 M $NH_4HCO_3$ buffer in µl (pH 8.5) | Isopropanol in µl | 20 mM GSH in µl | 4 mM GSSG in µl |
|---|---|---|---|---|---|
| 1 | 20 | 180 | - | - | - |
| 2 | 20 | 160 | - | 20 | - |
| 3 | 20 | 140 | - | 20 | 20 |
| 4 | 20 | 130 | 50 | - | - |
| 5 | 20 | 110 | 50 | 20 | - |
| 6* | 20 | 90 | 50 | 20 | 20 |
| 7 | 20 | 80 | 100 | - | - |
| 8 | 20 | 60 | 100 | 20 | - |
| 9 | 20 | 40 | 100 | 20 | 20 |
| 10 | 20 | 180 | - | - | - |

*condition 6 was used for stryphnine A oxidative folding.

Table S5. Amide co-efficients of stryphnine A.

| Residue | Amide proton temperature coefficient (ppb/K) |
|---|---|
| Cys1 | - |
| Leu2* | -2.9 |
| Pro3 | - |
| Lys4 | - |
| Gly5* | -3.4 |
| Thr6* | -1.2 |
| Pro7 | - |
| Cys8* | -2.5 |
| Pro9 | - |
| Ser10 | - |
| Thr11* | -3.3 |
| His12* | -2.4 |
| Pro13 | - |
| Tyr14 | -5.2 |
| Val15* | -1.8 |
| Cys16* | -1.6 |
| Cys17* | -2.8 |
| Ser18* | -4.4 |
| Gly19* | -3.5 |
| Ile20* | -1 |
| Cys21 | -8.2 |
| Tyr 22* | -2.4 |
| Gly23 | -7.2 |
| Val24 | -7.0 |
| Pro25 | - |
| Gly26 | - |
| Ala27* | -3.2 |
| His28 | - |
| Glu29 | -6.3 |
| Ile30* | -0.8 |
| Cys31* | -0.2 |
| Tyr32 | -5.1 |

*Amide proton temperature coefficient values (ppb/K) for stryphnine A were derived from variable temperature experiments (285-300 K). H protons with a temperature dependence of < -4.6 ppb/K are generally considered protected from the solvent with a high likelihood (85%) of being involved in hydrogen bonds as hydrogen bond donors.

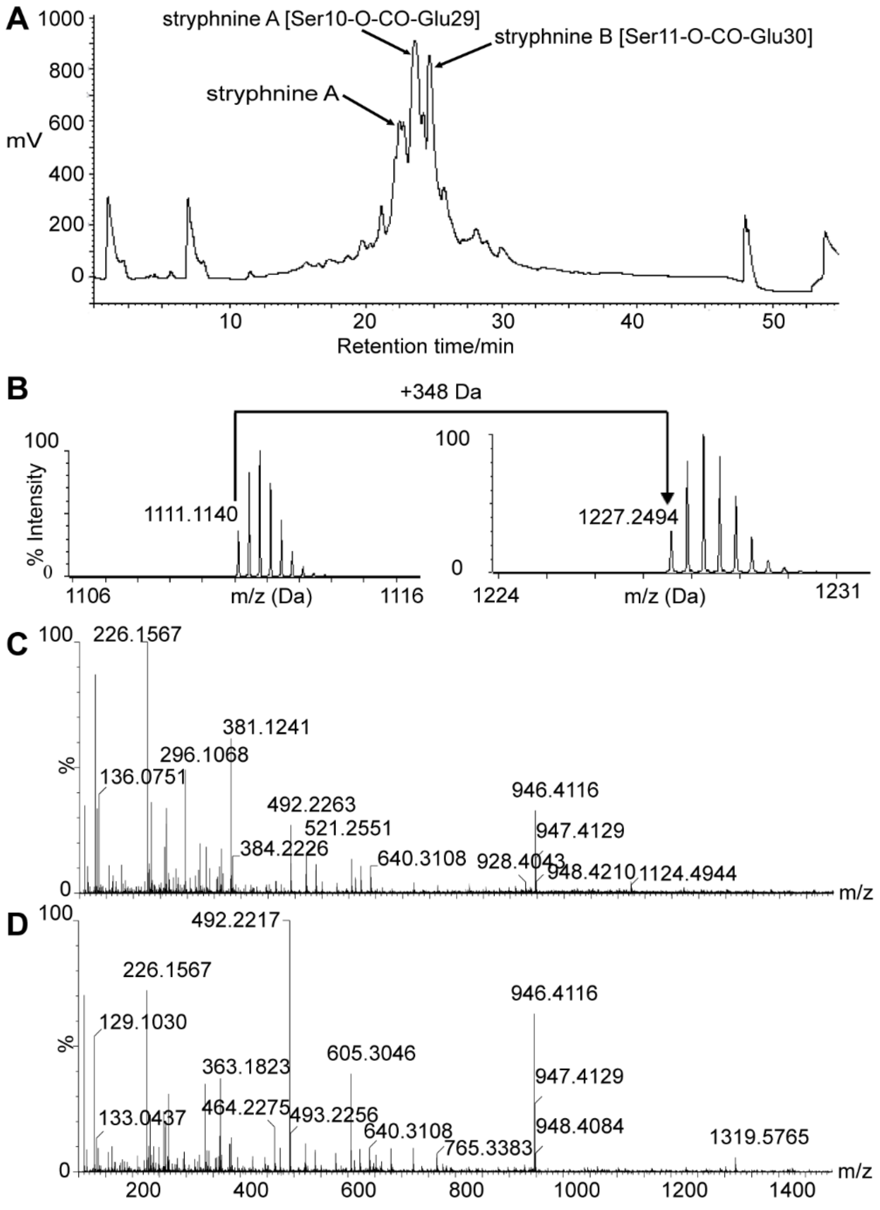


Fig. S1. Isolation of stryphnines from *S. fortis* and MS/MS sequencing of reduced/alkylated stryphnine A [Ser10-O-CO-Glu29] prior to enzymatic cleavage. **A**, RP-HPLC chromatogram of *S. fortis* 60% ACN extract. The peptides eluted in the following order of hydrophobicity, stryphnine A (22.5 min) < stryphnine A [Ser10-O-CO-Glu29] (23.0 min) < stryphnine B [Ser11-O-CO-Glu30] (26 min). **B**, Monoisotopic mass increment of the peptides by 348 Da after reduction and alkylation exemplified by stryphnine A [Ser10-O-CO-Glu29], indicated the presence of six cysteine residues. **C**, MS/MS spectra of reduced and alkylated stryphnine A [Ser10-O-CO-Glu29] **D,** MS/MS spectra of reduced and alkylated stryphnine A

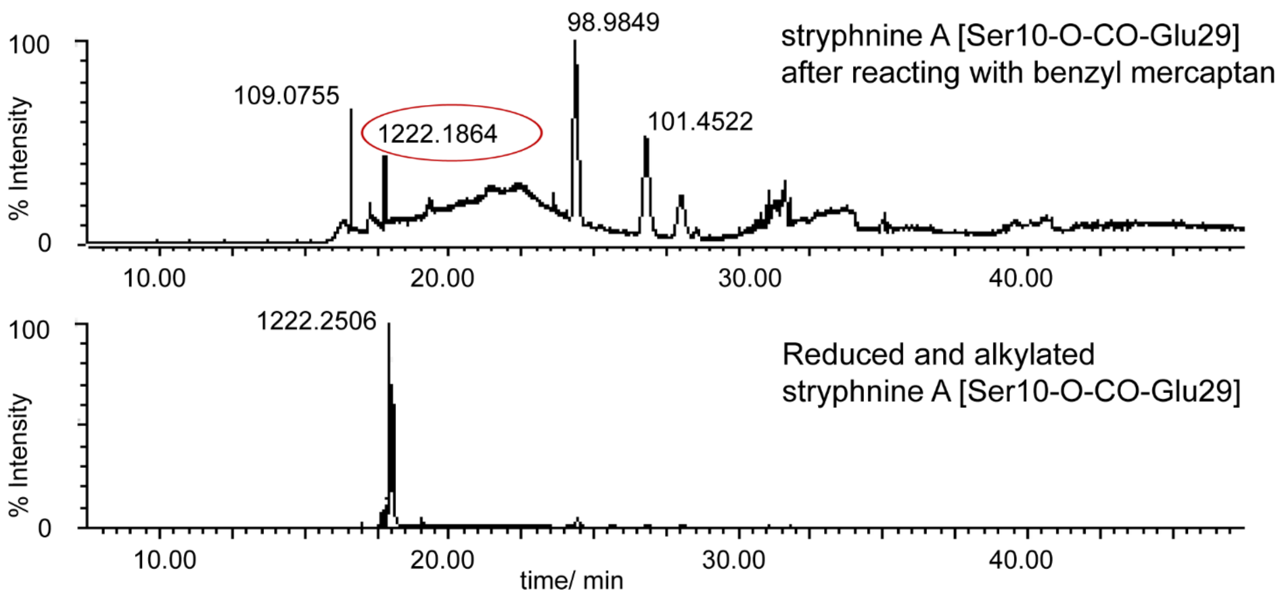


Fig. S2. Treatment of stryphnine A [Ser10-O-CO-Glu29] with benzyl mercaptan. No mass increment of stryphnine A [Ser10-O-CO-Glu29] after reacting with benzyl mercaptan indicated the absence of a dehydroalanine unit.

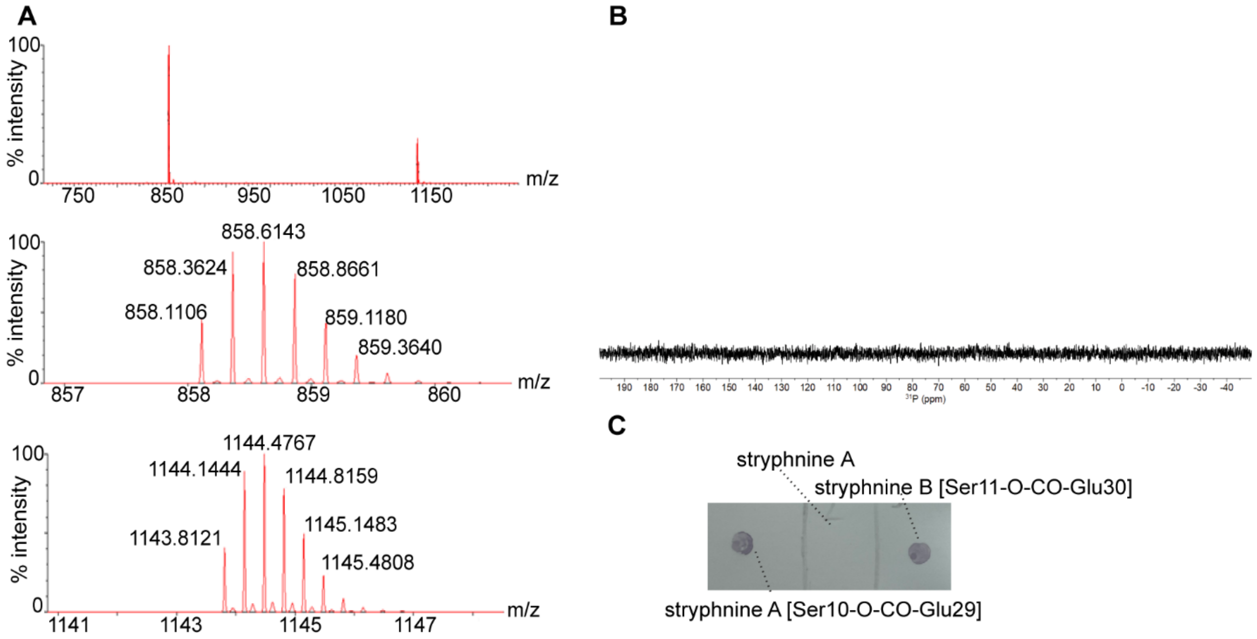


Fig. S3: Experiments conducted to rule out the presence of phosphorylation in stryphnines.
**A**, Synthetic stryphnine A incorporating a phosphorylated S10 showed a monoiosotopic m/z $[M+4H]^{4+}$ of 858.1106 indicating its ability to ionize well under MS conditions, confirming its different identity compared to native stryphnine A[Ser10-O-CO-Glu29]. **B**, The dot blot experiments using anti-phosphoserine antibodies was conducted to confirm the presence of phosphorylated serine in stryphnines. The spots corresponding to stryphnine A [Ser10-O-CO-Glu29] and stryphnine B [Ser11-O-CO-Glu30] resulted in binding of anti-phosphoserine antibodies, whereas no antibody binding was observed for stryphnine A. In the absence of phosphorylation, it appears that antibodies still bound the O-ester containing peptides due to structural similarity at the Ser-O-ester residue. **C**, 1D $^{13}P$ NMR spectrum of stryphnine A[Ser10-O-CO-Glu29] indicating the absence of phosphorylation.

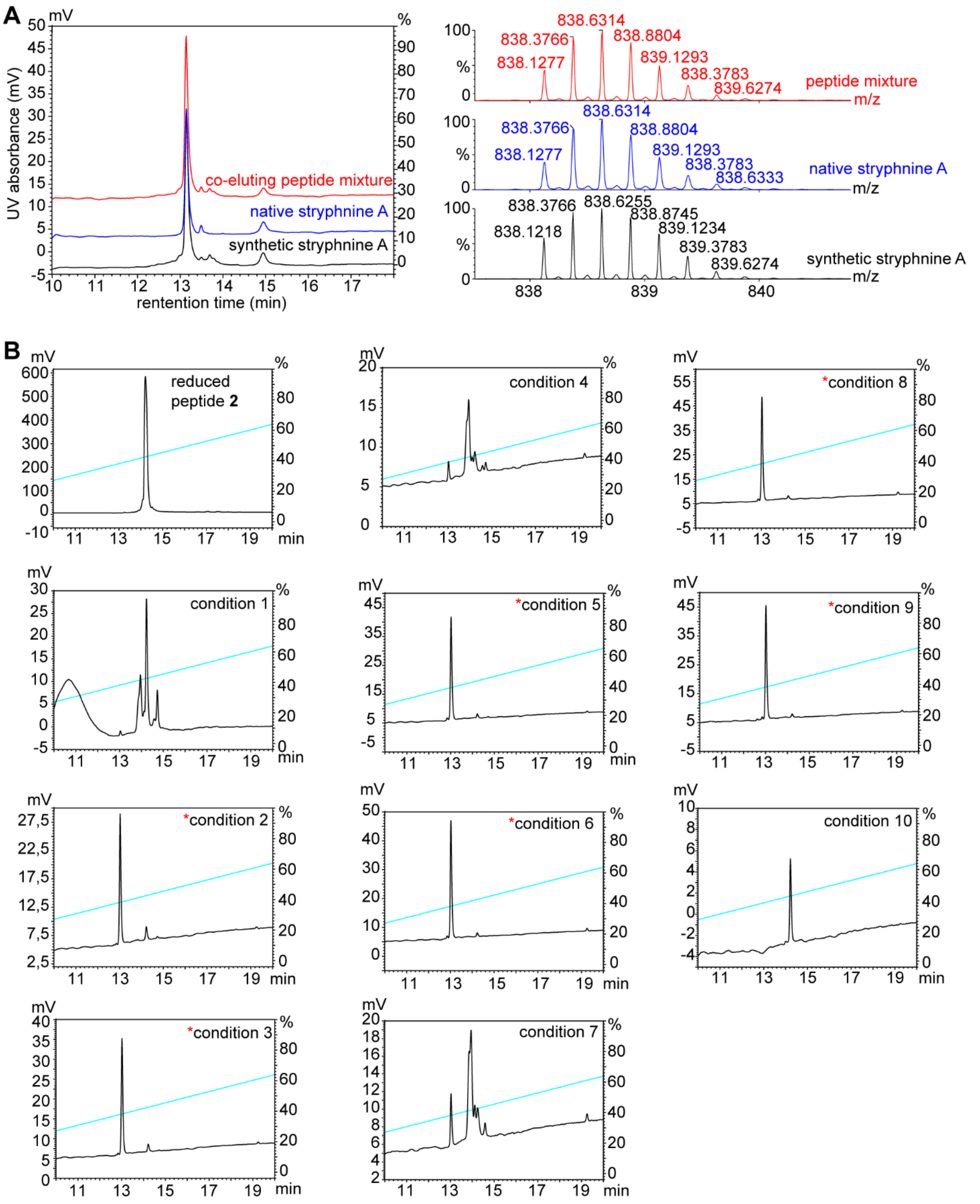


Fig. S4. Confirmation of the correct folding of synthetic stryphnine A. **A**, Co-elution of native and synthetic stryphnine A was confirmed by RP-HPLC and LC-MS analyses. **B**, Analytical RP-HPLC-UV spectra of synthetic stryphnine A under different oxidation conditions. An early eluting, prominent peak relative to the reduced peptide was observed in oxidation trials under where correct folding occurred, as marked by*.

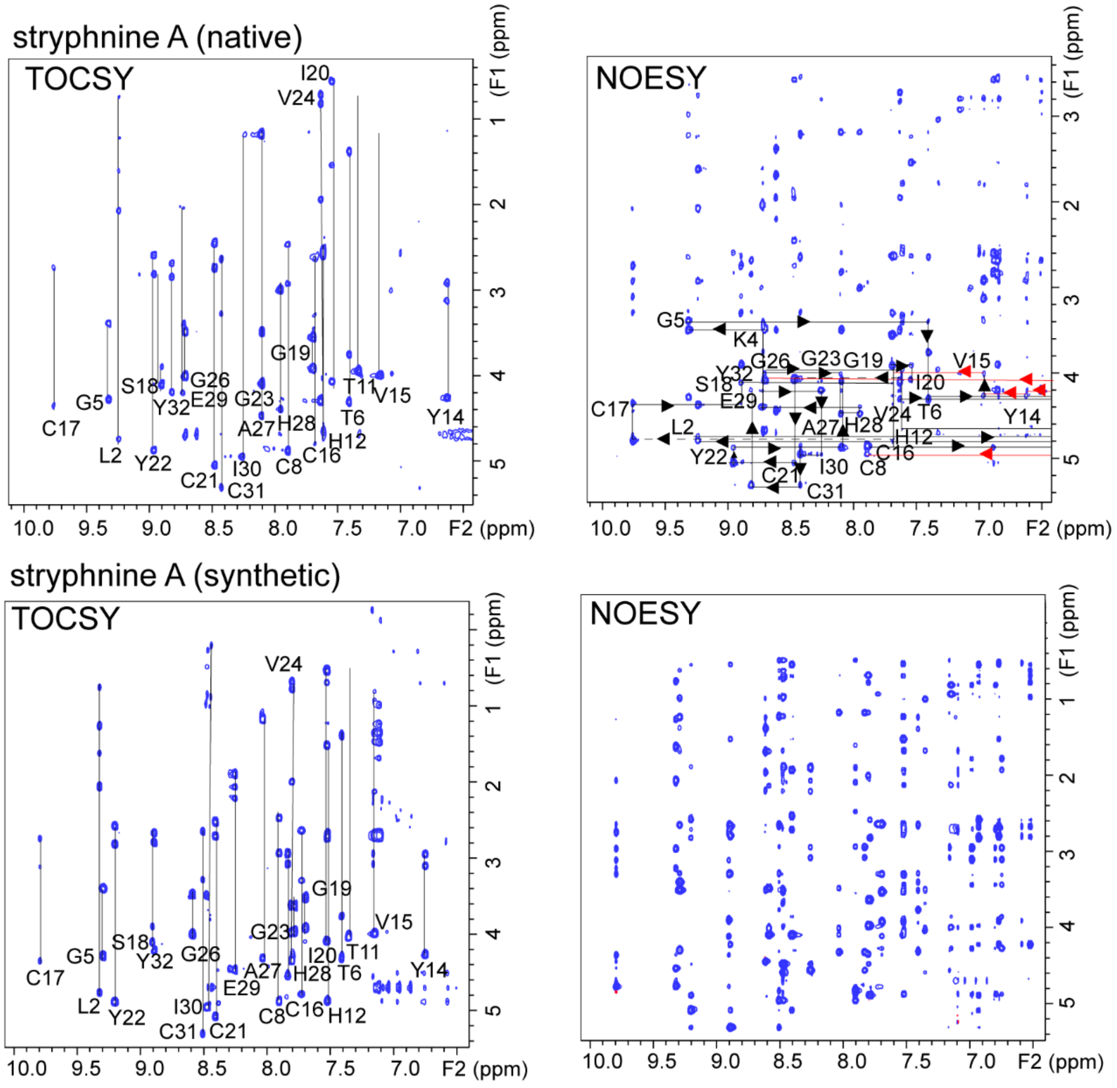


Fig. S5. Comparison of the fingerprint region of the TOCSY and NOESY spectra for native and synthetic stryphnine A. Individual spin systems were identified from the TOCSY data and NOESY data were used to link neighboring residues in the sequence. Sequential $\alpha H_i$-$NH_{i+1}$ NOEs were observed for the entire peptide chain except at Pro3, Pro7, Pro9, Pro13 and Pro25.

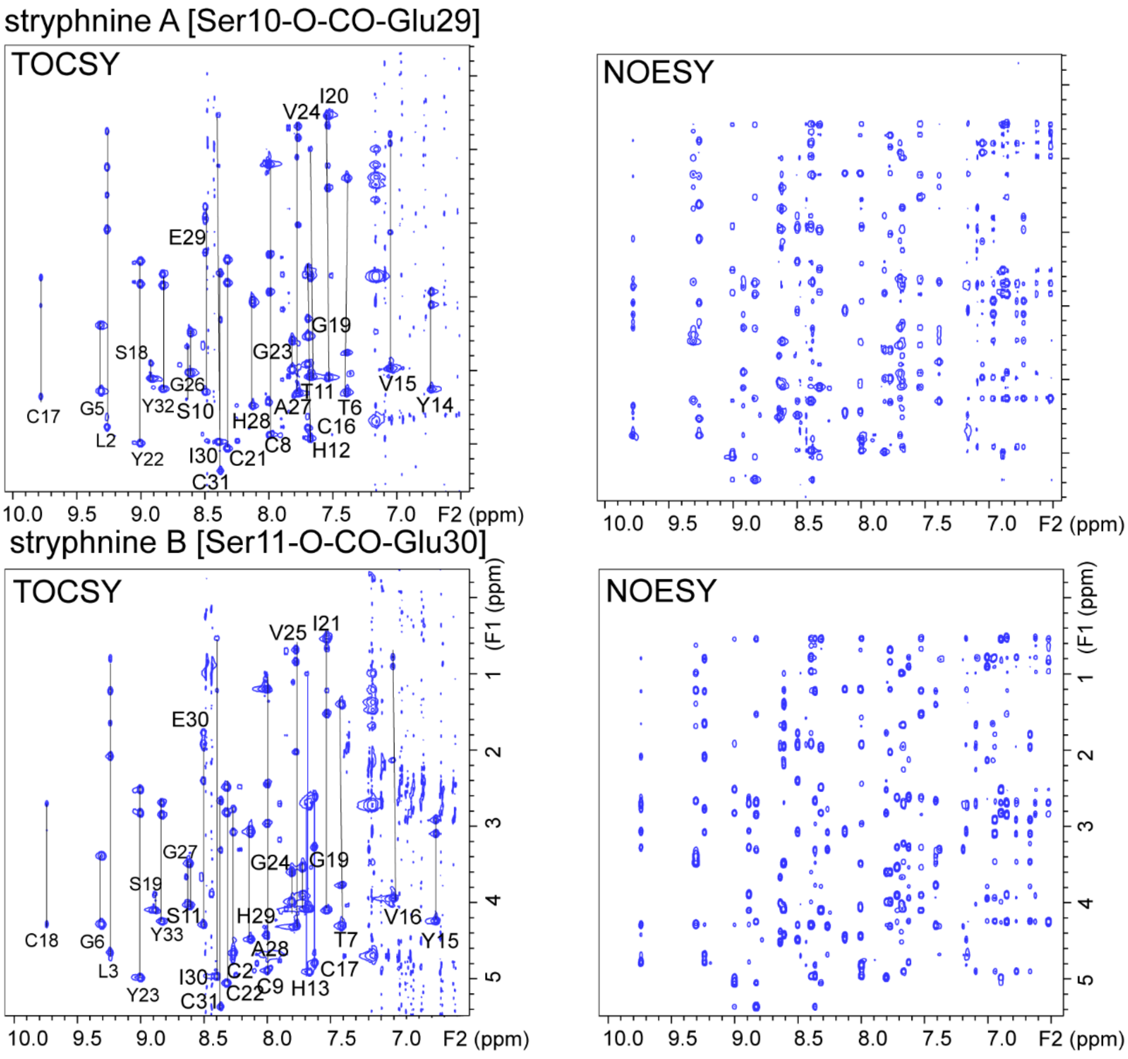


Fig. S6. Comparison of the fingerprint region of the TOCSY and NOESY spectra of stryphnine A [Ser10-O-CO-Glu29] and stryphnine B [Ser11-O-CO-Glu30]. Individual spin systems were identified from the TOCSY data and NOESY data were used to link neighboring residues in the sequence. Sequential $\alpha H_i$-$NH_i$+1 NOEs were observed for the entire peptide chain except at Pro4, Pro8, Pro10, Pro14 and Pro26.

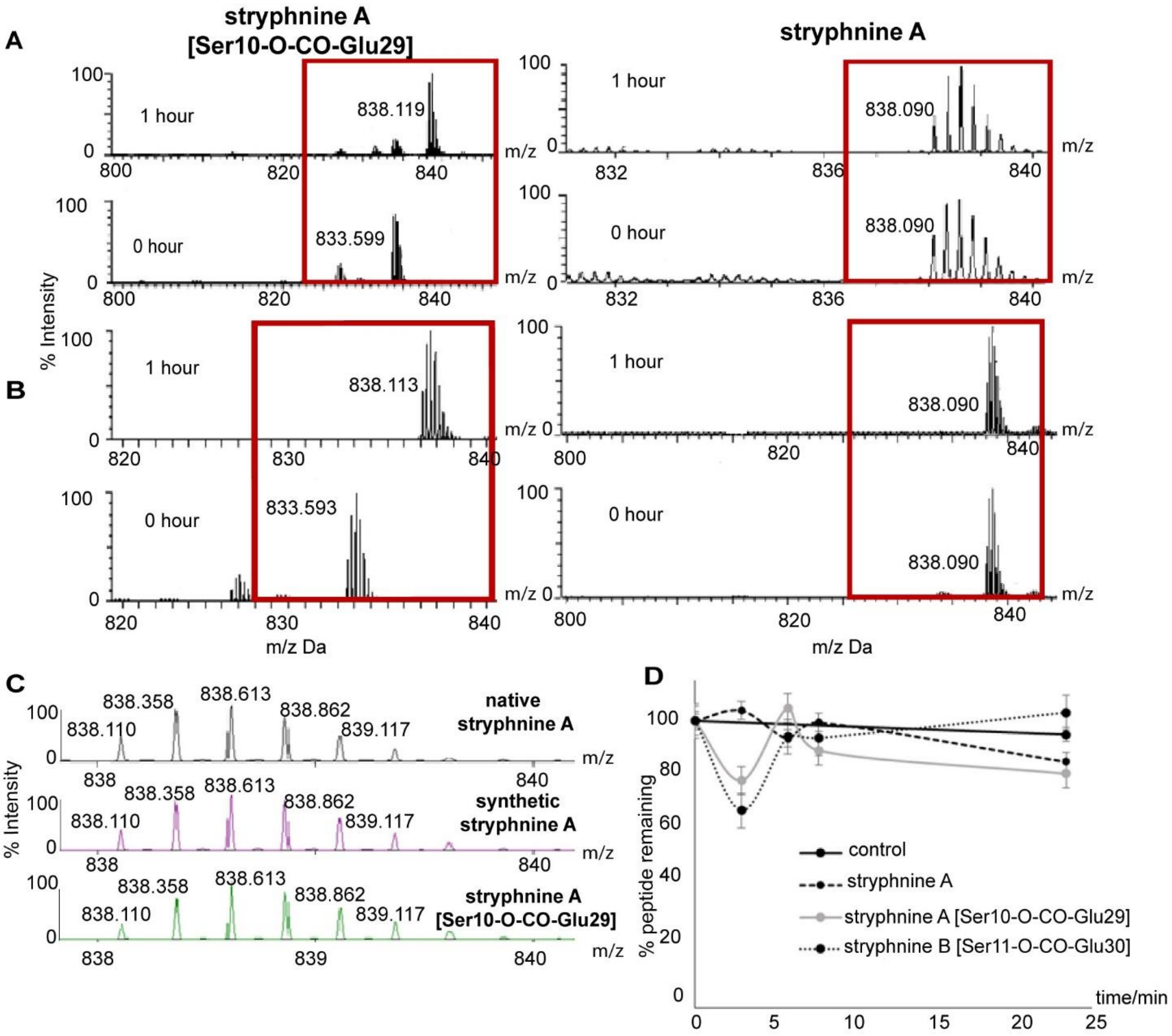


Fig. S7. Chemical, thermal, and human serum stabilities of stryphnines subjected to different pH treatments at elevated temperature (70 °C). **A**, Conversion of stryphnine A [Ser10-O-CO-Glu29] to stryphnine A after incubation for 1 h at pH 1 (left), No conversion of stryphnine A after incubation for 1 h at pH 1 (right). **B**, Conversion of stryphnine A [Ser10-O-CO-Glu29] to stryphnine A after incubation for 1 h at pH 7 (left), No conversion of stryphnine A after incubation for 1 h at pH 7 (right). **C,** The mass and ionization pattern of native stryphnine A, synthetic stryphnine A and stryphnine A [Ser10-O-CO-Glu29] after ester bond hydrolysis (incubation for 1 h at pH 8.5, 70°C). **D**, Stability of peptides in human serum. All stryphnines remained stable in serum at 37 °C with >80% peptide remaining and their intact mass detectable over a 24 h incubation period. Each point represents the means ± SDs of three experiments.

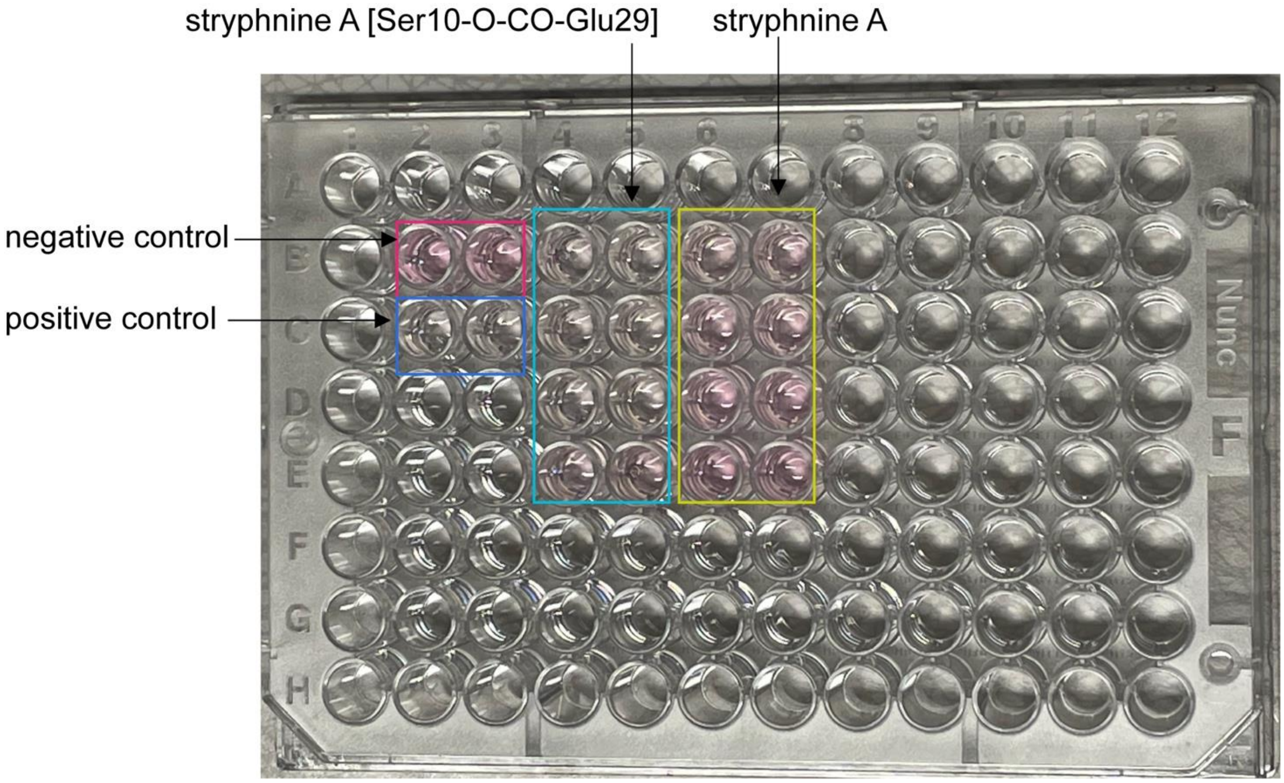


Fig. S8. Neuraminidase (Sialidase) inhibitory activity of the peptides.

This image was taken after 2 h incubation of the 96-well microtiter plate. Formation of resorufin, a red fluorescent compound indicated the absence of neuraminidase (sialidase) inhibitory activity of the peptides. The negative control wells (highlighted in pink color) indicated the absence of neuraminidase (sialidase) inhibitory due to the formation of resorufin. The positive control wells (highlighted in blue) were colorless due to the inhibition of neuraminidase (sialidase) enzyme. The wells highlighted in cyan color contained stryphnine A [Ser10-O-CO-Glu29] with the concentrations of 30, 21, 15 and 9 µM (top to bottom). At the top three concentrations, 30, 21 and 15 µM, the wells were colorless indicating an inhibition above 80% of neuraminidase (sialidase) enzyme. A weak activity appeared at 9 µM (57% inhibition) detectable by the slight red color. The wells highlighted in yellow contained stryphnine A, at concentrations of 60, 45, 30 and 15 µM (top to bottom). The red color appeared at all four concentrations. indicating the absence of strong neuraminidase

(sialidase) inhibitory activity. The red color intensity increased when reducing the concentration of stryphnine A.
